# The gut microbiota participates in the effect of linaclotide in patients with irritable bowel syndrome with constipation (IBS-C): a multicenter, prospective, pre-post study

**DOI:** 10.1186/s12967-024-04898-1

**Published:** 2024-01-23

**Authors:** Jianyun Zhou, Haoqi Wei, An Zhou, Xu Xiao, Xia Xie, Bo Tang, Hui Lin, Li Tang, Ruiping Meng, Xiaoying Yuan, Jing Zhang, Cheng Huang, Baobao Huang, Xiping Liao, Tingting Zhong, Suyu He, Sai Gu, Shiming Yang

**Affiliations:** 1 Department of Gastroenterology, The Second Affiliated Hospital, The Third Military Medical University, Xinqiaozheng Street, Chongqing, China 400037; 2 Department of Gastroenterology, The Ninth People’s Hospital of Chongqing, No. 69 Jialing Village, Beibei District, Chongqing, China 400700; 3 Department of Gastroenterology, Chongqing University Jiangjin Hospital, No.725, Jiangzhou Avenue, Jiangjin District, Chongqing, China 402260; 4 Department of Gastroenterology, Chonggang General Hospital, No. 1 Dayan Sancun, Dadukou District, Chongqing, China 400000; 5 Department of Gastroenterology, Suining Central Hospital, 22 Youfang Street, Chuanshan District, Suining, China 629000; 6 Department of Gastroenterology, The First Affiliated Hospital of Chongqing Medical University Jinshan Campus, 50 Jinyu Dadao, Liangjiang New District, Chongqing, China 401112

**Keywords:** IBS-C, Linaclotide, Gut microbiome, *Blautia*, SCFAs

## Abstract

**Background:**

Interindividual variation characterizes the relief experienced by constipation-predominant irritable bowel syndrome (IBS-C) patients following linaclotide treatment. Complex bidirectional interactions occur between the gut microbiota and various clinical drugs. To date, no established evidence has elucidated the interactions between the gut microbiota and linaclotide. We aimed to explore the impact of linaclotide on the gut microbiota and identify critical bacterial genera that might participate in linaclotide efficacy.

**Methods:**

IBS-C patients were administered a daily linaclotide dose of 290 µg over six weeks, and their symptoms were then recorded during a four-week posttreatment observational period. Pre- and posttreatment fecal samples were collected for 16S rRNA sequencing to assess alterations in the gut microbiota composition. Additionally, targeted metabolomics analysis was performed for the measurement of short-chain fatty acid (SCFA) concentrations.

**Results:**

Approximately 43.3% of patients met the FDA responder endpoint after taking linaclotide for 6 weeks, and 85% of patients reported some relief from abdominal pain and constipation. Linaclotide considerably modified the gut microbiome and SCFA metabolism. Notably, the higher efficacy of linaclotide was associated with enrichment of the *Blautia* genus, and the abundance of *Blautia* after linaclotide treatment was higher than that in healthy volunteers. Intriguingly, a positive correlation was found for the *Blautia* abundance and SCFA concentrations with improvements in clinical symptoms among IBS-C patients.

**Conclusion:**

The gut microbiota, especially the genus *Blautia*, may serve as a significant predictive microbe for symptom relief in IBS-C patients receiving linaclotide treatment.

*Trial registration*: This trial was registered with the Chinese Clinical Trial Registry (Chictr.org.cn, ChiCTR1900027934).

**Supplementary Information:**

The online version contains supplementary material available at 10.1186/s12967-024-04898-1.

## Background

Irritable bowel syndrome (IBS) is a pervasive, symptom-driven chronic disorder marked by abdominal discomfort and irregular bowel movements [1] that affects an estimated 11.2% of the global population [1, 2]. Approximately one-third of these patients are diagnosed with irritable bowel syndrome with constipation(IBS-C), a subtype of IBS. The therapeutic objectives primarily focus on alleviating symptoms, improving the patients’ quality of life, and reinstating their normal social functioning rather than eradicating the disease [3, 4]. Several guidelines and consensus statements have recommended the use of antidepressants, laxatives, prokinetics, and probiotics for the treatment of IBS-C patients. Due to an improved understanding of its pathogenesis, numerous therapeutic agents have been developed, and certain drugs have been removed from the front-line treatment strategies [5, 6]. Given the limitations of conventional treatments, there is mounting evidence highlighting the effectiveness of secretory drugs. These new drugs, which target chloride ion channels, have a clear mechanism of action [7, 8], and an example is the guanylate cyclase C agonist linaclotide [9, 10]. Linaclotide not only mitigates constipation symptoms but also ameliorates global symptoms such as abdominal discomfort, pain, and bloating [11–13]. This drug has been endorsed by the U.S. Food and Drug Administration (FDA) and the American College of Gastroenterology for the treatment of chronic idiopathic constipation (CIC) and IBS-C in adults [3, 14].

However, the efficacy of linaclotide treatment has been shown to exhibit significant interindividual variation [15]. A 2018 phase III trial in China revealed that approximately 40% of patients did not respond to linaclotide treatment, potentially due to the multifactorial etiology of IBS-C [16]. To date, the causes of IBS-C have not been fully elucidated, and previous research has focused on alterations in the intestinal flora that are intimately connected with IBS-C [17]. Studies have suggested that the gut microbiota influences not only the onset of IBS-C but also the effectiveness of disease treatment, including the chemotherapy sensitivity in colon cancer patients [18], the impact of metformin on diabetes [19], and the efficacy of rifaximin in treating IBS-D [20]. Through animal experiments, a Japanese study confirmed that linaclotide can enhance the intestinal milieu in patients with renal insufficiency [21]. However, whether linaclotide can ameliorate gut microflora dysbiosis and whether the intestinal flora is correlated with symptom relief in linaclotide-treated IBS-C patients remains to be established.

Thus, the potential influence of the gut microbiota on the therapeutic effects of linaclotide has significant implications for IBS-C treatment. Through a multicenter clinical trial, this study aimed to evaluate the efficacy and safety of linaclotide and discern the relationship between the relief provided by linaclotide treatment and alterations in the gut microbiota.

## Methods

### Subjects

This multicenter, pre-post clinical trial, which spanned a treatment period of six weeks, was conducted at six Chinese hospitals between January 2020 and June 2021 (Additional file 7: Table S1). The inclusion criteria for IBS-C patients were the following: ① age > 18 years, ② diagnosis of IBS-C (Rome IV diagnostic criteria), ③ Bristol type 1 or 2 > 25% [22], and ④ < 3 bowel movements per week. The exclusion criteria were as follows: ① pregnant or lactating women; ② patients with mental illness; ③ patients who consumed probiotics or antibiotics one month prior to the study; ④ patients who had undergone intestinal cleansing in the past two weeks; ⑤ patients allergic to the drugs used in the study; ⑥ patients with a history of digestive system tumors or gastrointestinal surgery, intestinal obstruction or gastrointestinal bleeding; and ⑦ patients with severe heart or lung diseases.

The exclusion criteria for the healthy volunteers were as follows: ① age < 18 years; ② use of antibiotics, probiotics, bowel cleansing products or proton-pump inhibitors within 1 month prior to the study; and ③ diagnosis of diseases such as IBS, inflammatory bowel disease, coeliac disease, or digestive system tumors or history of gastrointestinal surgery, intestinal obstruction or gastrointestinal bleeding, cardiac, renal or hepatic diseases, metabolic diseases, lactose intolerance, or active infection with pathogenic microorganisms.

### Treatment and follow-up

All patients received the same dosage of linaclotide (290 μg orally once a day, half an hour before breakfast) for 6 weeks. The medication was supplied by AstraZeneca. All patients were followed up twice a week for 10 weeks post enrollment. The patients were not allowed to take any other constipation-related drugs, including probiotics and laxatives, during the treatment period. For patients with other diseases, treatment could be administered according to the established guidelines, with the requirement of keeping objective records.

### Collection of fecal samples

Fecal samples were collected before and after 6 weeks of linaclotide treatment. All samples were collected using a special stool collection tube in the hospital, and samples that did not come into any obvious contact with the external environment were collected with a stool shovel. Three stool samples (each consisting of at least 1 *g*) were collected from each patient, placed in a special refrigerator for specimen collection, stored at – 20 ℃, and rapidly transferred to − 80 ℃ by laboratory personnel within 4 h [23].

### Indicator collection

The changes in abdominal pain (numeric rating scale (NRS), gastrointestinal symptom rating scale (GSRS), symptom severity score of irritable bowel syndrome (IBS-SSS), and quality of life scale of irritable bowel syndrome (IBS-QoLS)) were evaluated biweekly for 10 weeks, and the Bristol stool form scale (BSFS) and spontaneous bowel movements (SBMs) were assessed daily during treatment based on dietary conditions [24]. Drug-related side effects were also monitored. Fecal samples were collected before and 6 weeks after linaclotide administration. All these indicators were collected by trained personnel responsible for data collection at each center, and the organizing unit inspected and reassessed the data weekly. After treatment, the patients were categorized into relief and no relief groups. The patients in the relief group were further divided into responders and nonresponders based on the FDA response criteria [25].

The FDA response endpoint criteria for IBS-C were as follows: ① ≥ 30% reduction from baseline in the weekly mean of the daily scores for abdominal pain; ② ≥ 1 increase in the CSBM per week from baseline; and ③ improvement in abdominal pain and CSBM in the same week during at least 50% of the treatment period.

The relief criterion for IBS-C was the alleviation of abdominal pain or constipation during at least 50% of the treatment period.

### 16S rRNA sequencing

DNA extraction, PCR amplification, fluorescence quantification, MiSeq library construction and MiSeq sequencing were subsequently performed. PE reads obtained by MiSeq sequencing were first spliced according to overlap, and the sequence quality was then controlled and filtered. Operational taxonomic unit (OTU) clustering analysis and species taxonomy analysis were performed after sample segmentation. Multiple diversity indices could be analyzed based on the OTU clustering analysis results and the detected sequencing depth. Using taxonomic information, the community structure was statistically analyzed at each taxonomic level. Based on the abovementioned analysis, a series of in-depth statistical and visual analyses, including multivariate analysis and significance tests, were conducted to analyze the community composition and phylogenetic information of diverse species.

### Metabolomics testing

Appropriate amounts of pure standards of short-chain fatty acids, including acetic acid, propionic acid, butyric acid, isobutyric acid, valeric acid, isovaleric acid and hexanoic acid, were used. Ten standard concentration gradients (0.02, 0.1, 0.5, 2, 10, 25, 50, 100, 250, and 500 μg/ml) were formulated with ether. An appropriate amount of sample was placed into a 2-ml centrifuge tube, and 50 μl of 15% phosphoric acid, 100 μl of 125 μg/ml internal standard (isohexic acid) solution, and 400 μl of ether were then added. After homogenization for 1 min, centrifugation was performed at 12,000 RPM at 4 ℃ for 10 min, and the supernatant was collected for testing. Appropriate chromatographic and mass spectrometry conditions were used. The precision, repeatability, and recovery were within a reasonable range.

### Statistical methods

The appropriate statistical analysis for comparisons between groups was selected based on the data distribution and patient characteristics, and the analyses were performed using GraphPad Prism 8.0 and IBM SPSS Statistics 26 software. The alpha diversity was determined by sampling-based OTU analysis, and the beta diversity was visualized and tested by principal coordinate analysis (PCoA) plots and analysis of similarities (ANOSIM). Linear discriminant analysis (LDA) was employed to analyze the differences in the bacterial community predominance between groups. Correlation analyses were performed using Spearman’s correlation. The data are presented as the means ± SDs, medians (P25-P75 values), medians (mix-max values), and n (%) values, and the significance was marked as follows: *p < 0.05, **p < 0.01, ***p < 0.001, ****p < 0.0001, and NS for no significance.

## Results

### Patient demographics

**Fig. 1:**
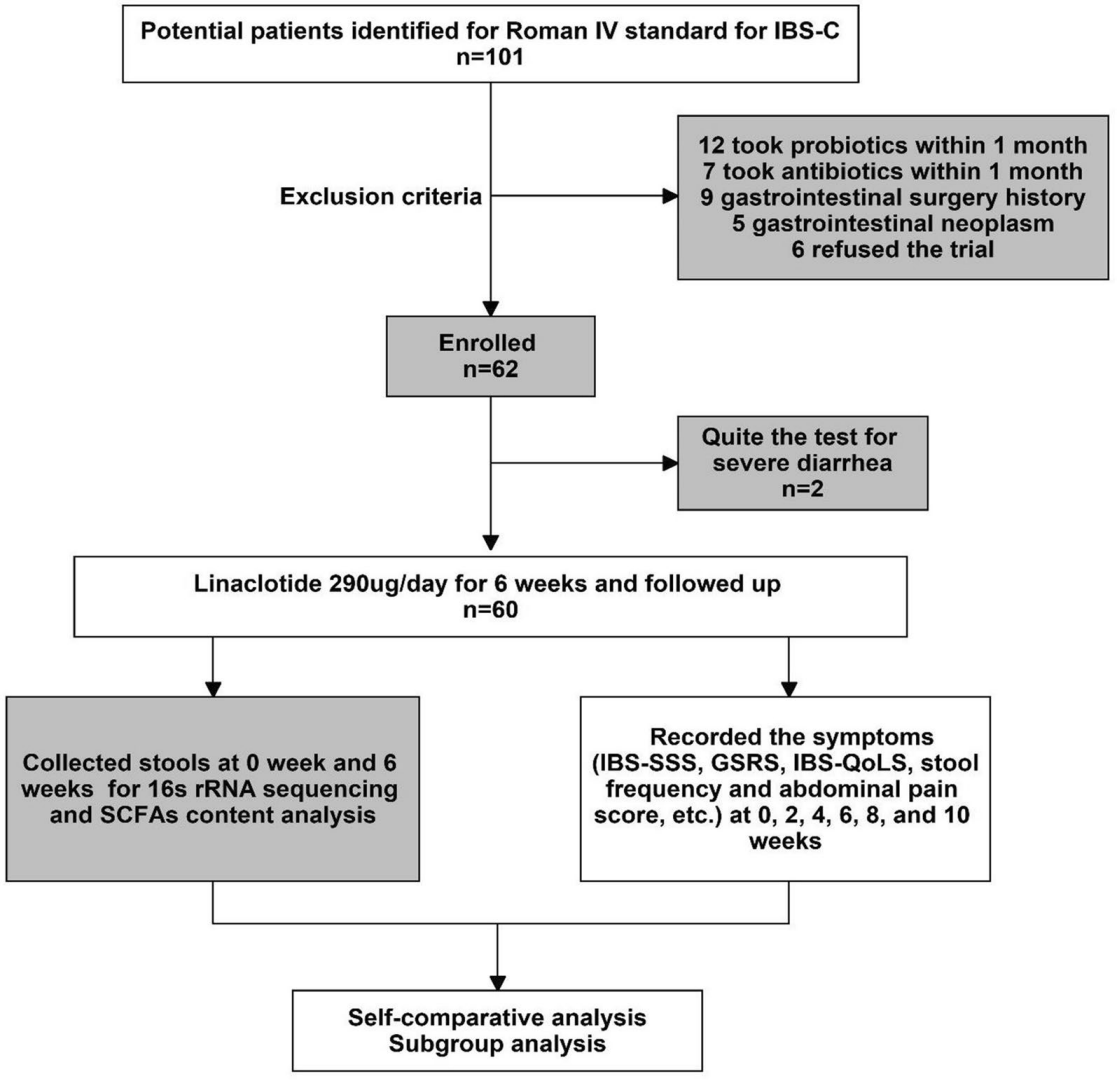
Clinical trial flow chart

A total of sixty-two patients diagnosed with IBS-C were recruited for the study between January 2020 and June 2021. Two patients (3.2%) discontinued participation due to severe diarrhea (Fig. 1). Among the recruited IBS-C patients, 86.7% were female, and the average age, IBS-SSS score, and body mass index (BMI) of the patients were 45.2 ± 10.97 years, 225.17 ± 92.296, and 22.62 ± 2.76, respectively (Table 1). No significant differences in age, BMI, education, underlying diseases, or drug-related side effects were observed between the patients who experienced relief and those who did not (P > 0.05). Prior to linaclotide therapy, no significant differences in the IBS-SSS scores were found between the two groups (Table 1). Concurrently, a cohort of thirty healthy volunteers was enrolled, and no notable differences in baseline characteristics were observed between the group of volunteers and the IBS-C patient group (Additional file 8: Table S2). To mitigate the potential confounding effects of dietary factors on the gut microbiota, we assessed the nutrient intake of in IBS-C patients using a food frequency questionnaire (FFQ) [26], and no significant differences were found in the total calorie, protein, fat, fiber, or carbohydrate intake from baseline to week 10 (Additional file 9: Table S3).

**Table 1:** The demographics of patients

		Total (N = 60)	Non-relief (n = 9)	Relief (n = 51)	P value
Gender (n,%)	Female	52 (86.7)	9 (100)	43 (84.31)	0.024
	Male	8 (13.3)	0 (0)	8 (15.69)	
Age	Median (P25-P75)	47 (36.5 – 51.75)	40 (33 – 50.5)	47 (38 – 53)	0.722
	Mean ± SD	45.2 ± 10.97	42.89 ± 10.96	45.61 ± 11.16	
BMI	Median (P25-P75)	22.48 (21.26 – 23.99)	21.78 (18.598 – 23.05)	22.58 (21.34 – 24.28)	0.100
	Mean ± SD	22.62 ± 2.76	20.94 ± 3.3	22.92 ± 2.75	
Education	Primary	6 (10)	0 (0)	6 (11.76)	0.495
	Medium	32 (53.3)	7 (77.78)	25 (49.02)	
	Higher	22 (36.7)	2 (22.22)	20 (39.22)	
IBS-SSS	Median (P25-P75)	225 (156.25 – 275)	225 (162.5 – 342.5)	225 (125 – 275)	0.445
(Baseline)	Mean ± SD	225.17 ± 92.296	245.56 ± 86.05	221.57 ± 86.89	
Basic disease *	No	56 (93.3)	9 (100)	47 (92.16)	0.347
	Yes	4 (6.7)	0 (0)	4 (7.84)	
Drug-related side effects ^#^	No	44 (73.3)	8 (88.89)	36 (70.59)	0.360
	Yes	16 (26.7)	1 (11.11)	15 (29.41)	

*BMI* body mass index, *IBS-SSS* irritable bowel syndrome symptom severity scale

^*^4 reported hypertension; # 16 reported diarrhea

### Clinical efficacy of linaclotide

**Fig. 2:**
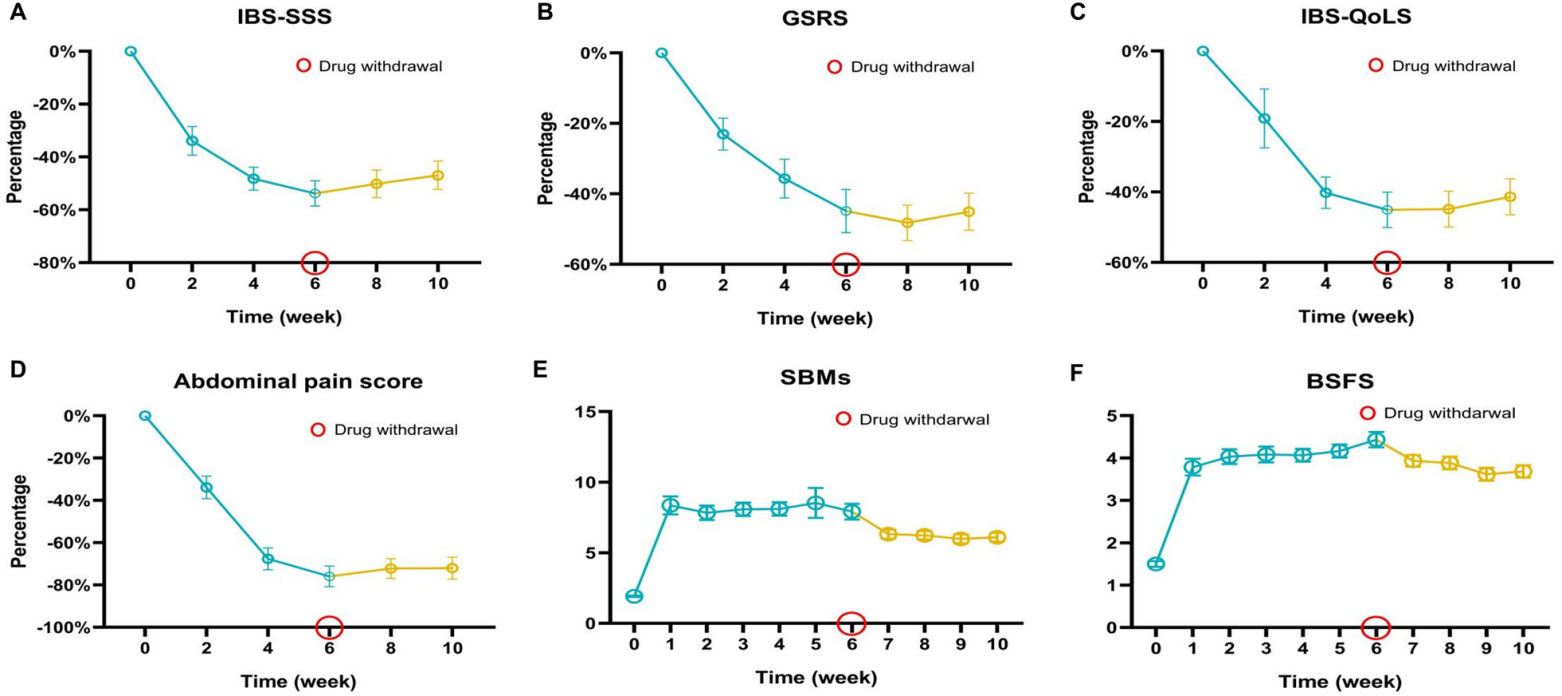
Changes in symptom scores during 6 weeks of treatment and 4 weeks of withdrawal. The irritable bowel syndrome symptom severity scale (IBS-SSS) (**A**), gastrointestinal symptom rating scale (GSRS) (**B**), irritable bowel syndrome quality of life questionnaire (IBS-QoLS) (**C**), and abdominal pain score (**D**) significantly decreased during 6 weeks of treatment, and these scores remained stable for four weeks after withdrawal. The Bristol Stool Form Scale (BSFS) (**E**) and spontaneous bowel movements (SBMs) (**F**) improved during the treatment and drug withdrawal periods

To evaluate the impact of linaclotide, we collected relevant clinical data (see the supplementary data and patient information table for detailed information). The IBS-SSS, GSRS, IBS-QoLS, and abdominal pain scores gradually decreased over six weeks compared with the baseline scores (Fig. 2A-D). The BSFS scores and number of SBMs progressively increased during the six weeks of treatment (Fig. 2E, F. After the six-week medication period, we conducted a four-week follow-up. During this period, the IBS-SSS, GSRS, IBS-QoLS, abdominal pain, BSFS, and SBM scores of the patients remained stable. By applying the FDA response endpoint criteria, we determined that 43.3% (26/60) of patients achieved the FDA response endpoint. Eighty-five percent (51/60) of the patients reported some relief. Among the patients who did not meet the FDA response criteria, 73.5% (25/34) reported at least some alleviation of abdominal pain and constipation with clinical relief, whereas 26.5% (9/34) reported no relief over six-week treatment course. These findings highlight the interindividual variation in the therapeutic efficacy of linaclotide.

### Linaclotide altered the intestinal flora of IBS-C patients

**Fig. 3:**
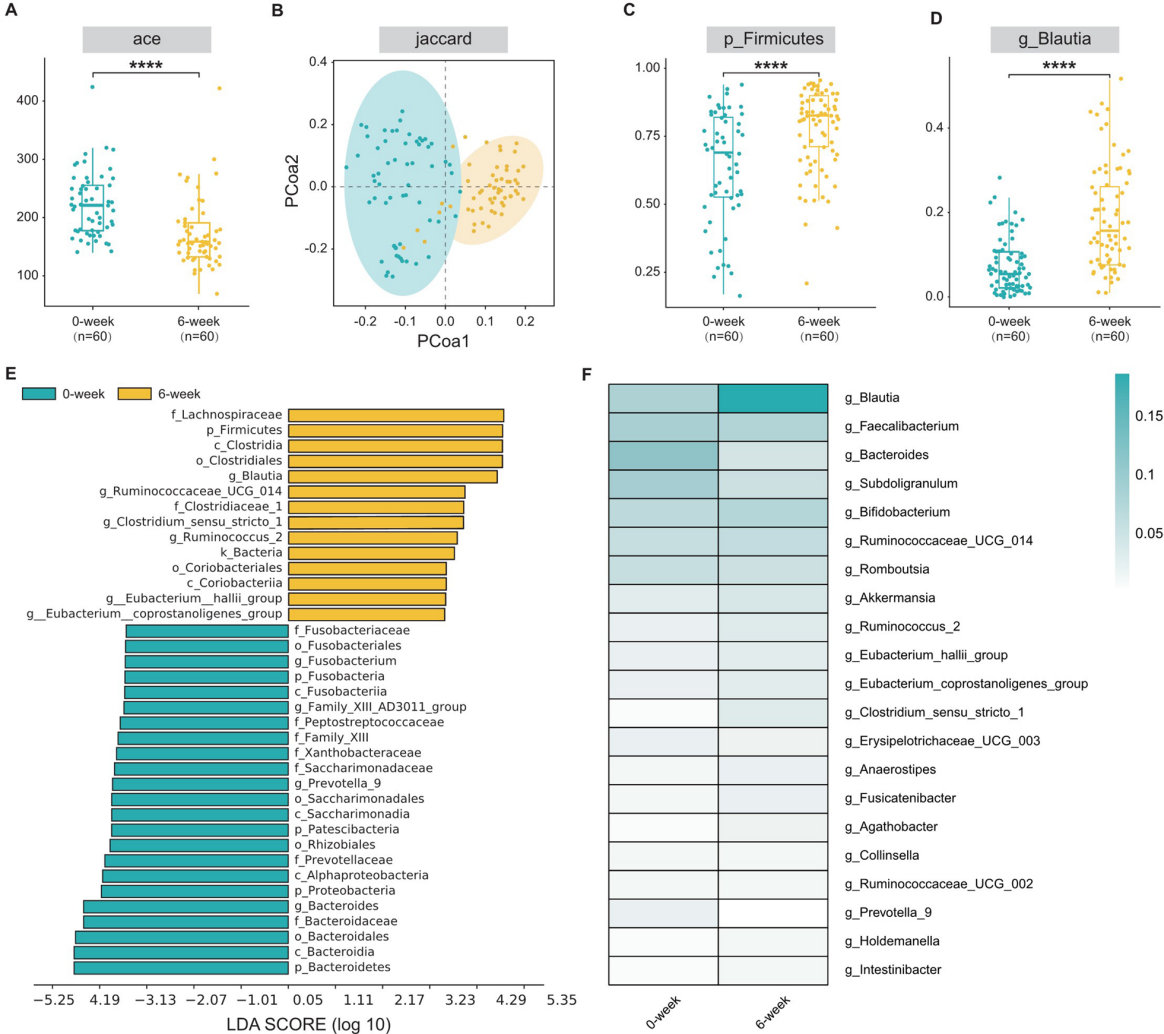
The efficacy of linaclotide in the treatment of IBS-C was related to its ability to modulate the gut microbiota. **A** Changes in alpha diversity indices. **B** Principal coordinate analysis (PCoA) of the gut microbiota. **C**, **D** The abundance of *Firmicutes* at the phylum level and that of *Blautia* at the genus level were greater after 6 weeks of treatment than at 0 weeks. **E** Linear discriminant analysis (LDA) was used to estimate the impact of the abundance of each component (genus) on the effects. **F** Community heatmap generated using color gradients to represent the size of the data in a two-dimensional matrix or table and to present community species composition information. ACE, Jaccard, and the abundances of *Firmicutes* and *Blautia* were adjusted for the IBS-SSS baseline score, sex, age, BMI, education, basic disease status, and nutrient status by repeated-measures ANOVA

To investigate whether the effect of linaclotide treatment was associated with changes in the gut microbiota, we collected initial (0-week) and final (6-week) fecal samples from 60 patients and performed high-throughput gene sequencing analysis of 16S rRNA to analyze the gut microbial composition. We assessed the alpha diversity of the gut microbiota using various methodologies through a generalized linear model, and consistent results across different indices (ACE and Chao1) revealed that the 6-week group exhibited significantly higher alpha diversity than the 0-week group (p < 0.0001; Fig. 3A, Additional file 10: Table S4). To further elucidate the differences in the gut microbial composition, we evaluated the beta diversity through principal coordinate analysis (PCoA) using the Jaccard distance algorithm. Clear clustering separation of operational taxonomic units (OTUs) indicated distinct community structures between the 0-week and 6-week groups, indicating significant differences in the structures (Fig. 3B).

Subsequently, we conducted a comprehensive examination of the gut microbiota landscape in all available samples to further explore the potential composition differences between the 0-week and 6-week groups. At the phylum level, *Firmicutes* constituted the most abundant phylum, accounting for 63.81% and 77.93% of the gut microbial community in the 0-week and 6-week groups, respectively (p < 0.0001) (Fig. 3C, Additional file 11: Table S5). Notably, at the genus level, distinct differences in the biological composition were observed between the two groups. We analyzed bacterial genera with a relative abundance exceeding 1%. Among them, the genera *Blautia* (18.57% vs. 7.77%, respectively; p < 0.001) (Fig. 3D, Additional file 12: Table S6) and *Fusicatenibacter* (1.23% vs. 1.69%, respectively; p < 0.001) (Additional file 12: Table S6) exhibited relatively higher abundances in the 6-week group. We also compared the taxonomic compositions at the class/order/family level between the two groups (Additional file 1: Figure S1B-D, Additional file 1,3: Table S7, Additional file 1,4: Table S8, Additional file 1,5: Table S9) and detected significant differences in the abundances of *Lachnospiraceae*, *Clostridiales* and *Clostridia*, to which *Blautia* belong, between before and after treatment.

To confirm the specific bacterial taxa affected by linaclotide treatment, linear discriminant analysis of effect size (LEfSe) was used for high-dimensional class comparisons, revealing significant differences in the predominance of the bacterial communities between the two groups (Fig. 3E, Additional file 2: Figure S2A). According to the results, the genus *Blautia* (from the phylum *Firmicutes* and the family *Lachnospiraceae*) emerged as a key bacterial type contributing to gut microbiota dysbiosis in the 6-week group. Additionally, a heatmap comparing the gut microbiota between the two groups based on the OTU abundance was generated at the genus level, further demonstrating the relatively higher abundance of the genus *Blautia* in the 6-week group, which aligned with the findings from the LEfSe analysis (Fig. 3F).

**Fig. 4:**
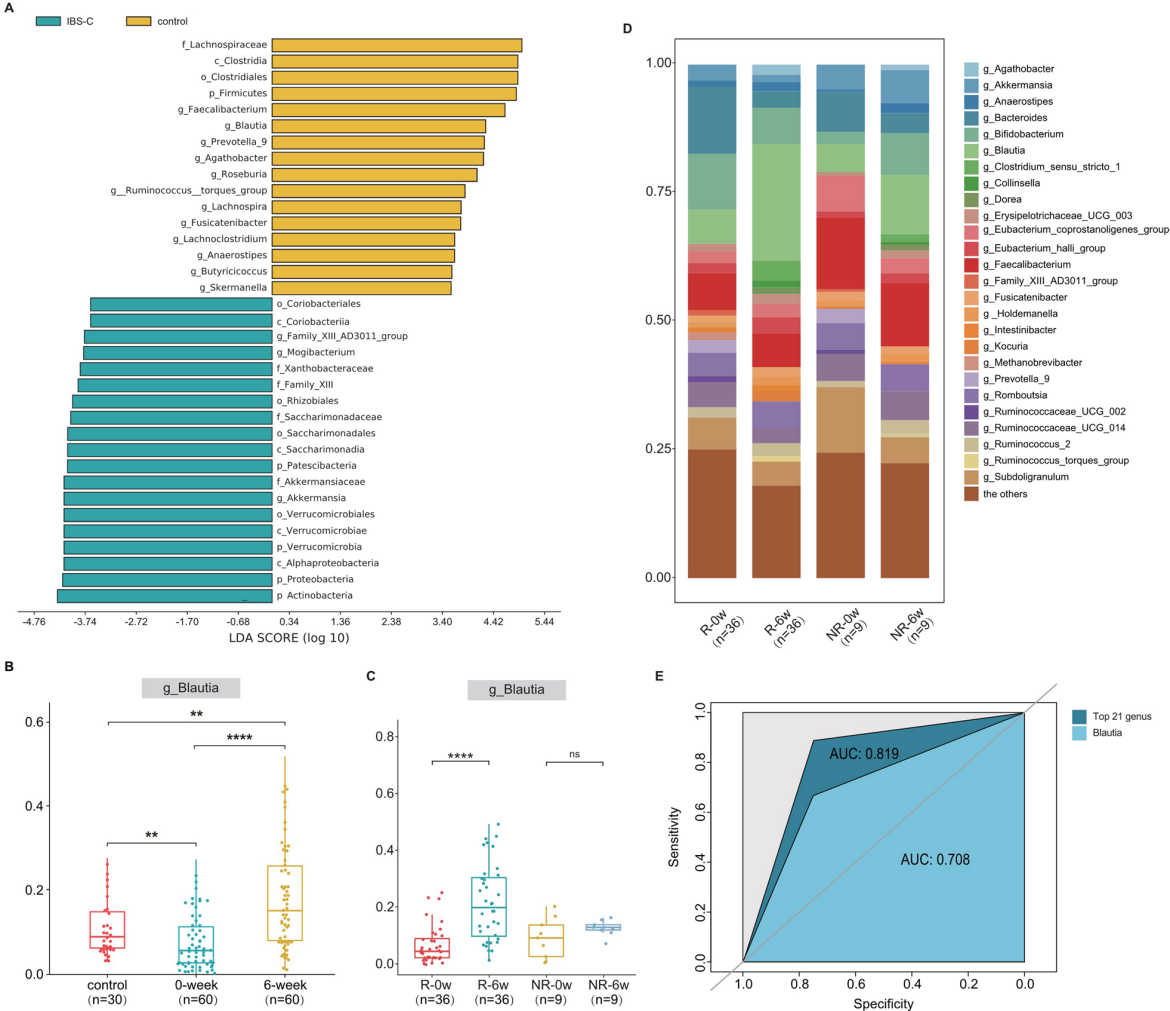
Linaclotide mitigated IBS-C in individual patients to different extents, and the detected increase in the abundance of *Blautia* was effective at alleviating the symptoms of IBS-C caused by linaclotide. **A** Linear discriminant analysis (LDA) of effect size (LEfSe) was used to estimate the impact of the abundance of each component on the different effects between IBS-C patients and control individuals (healthy volunteers). **B** The abundance of *Blautia* at the genus level was highest in the 6-week group, followed by the control group (healthy volunteers), and the 0-week group had the lowest abundance. **C**, **D** Abundance of *Blautia* in the relief and no-relief patients before and after treatment. **E** Receiver operating characteristic (ROC) curve for the top twenty-one bacteria at the genus level and *Blautia* for separate prediction; the area under the curve (AUC) is shown

Moreover, we analyzed the differences in the gut microflora between healthy volunteers and IBS-C patients. The LEfSe results indicated that *Firmicutes* at the phylum level and *Blautia* at the genus level were predominant in the healthy volunteers (Fig. 4A). In contrast, IBS-C patients exhibited a lower abundance of *Blautia* than did healthy volunteers, whereas the abundance of *Blautia* after linaclotide treatment was higher than that of the healthy volunteers (Fig. 4B). In addition, in the continuous observation (4 weeks after linaclotide withdrawal), we also found that *Blautia* remained higher than pretherapy, but lower than the period of treatment (Additional file 3: Figure S3). These findings further support the notion that linaclotide may exert its effects by modulating the intestinal flora.

### The abundance of *Blautia* was related to the efficacy of linaclotide

A relationship between the abundance of *Blautia* and the effectiveness of linaclotide treatment was observed in this study. To control for confounding factors such as sex, age, and BMI, a ratio of 1:4 was used to match 9 patients who did not experience relief (NR) and 36 out of 51 patients who reported relief [27]. Stool specimens collected at 0 and 6 weeks were also analyzed (Additional file 4: Figure S4A-E), which demonstrated that the overall structure and alpha diversity of the gut microbiota did not differ between the relief and no relief patients (Additional file 5: Figure S5A, B). Interestingly, more pronounced symptom relief was observed if the *Blautia* abundance after treatment was markedly higher than that before treatment (Fig. 4C, D. We also investigated the predictive ability of the intestinal flora by receiver operating characteristic (ROC) analysis and found that the baseline abundance at the genus level could be used to predict the efficacy of the IBS-C treatment with an area under the curve (AUC) of 0.819, and *Blautia* (AUC of 0.708) was among the top 21 genera (Fig. 4E). These findings suggested that some specific gut microbes, such as *Blautia*, exhibited a significant (p < 0.01) difference in their impact on the effect of linaclotide between patients who experienced relief and those who did not.

### The *Blautia* and SCFA levels were positively correlated with the alleviation of clinical symptoms

**Fig. 5:**
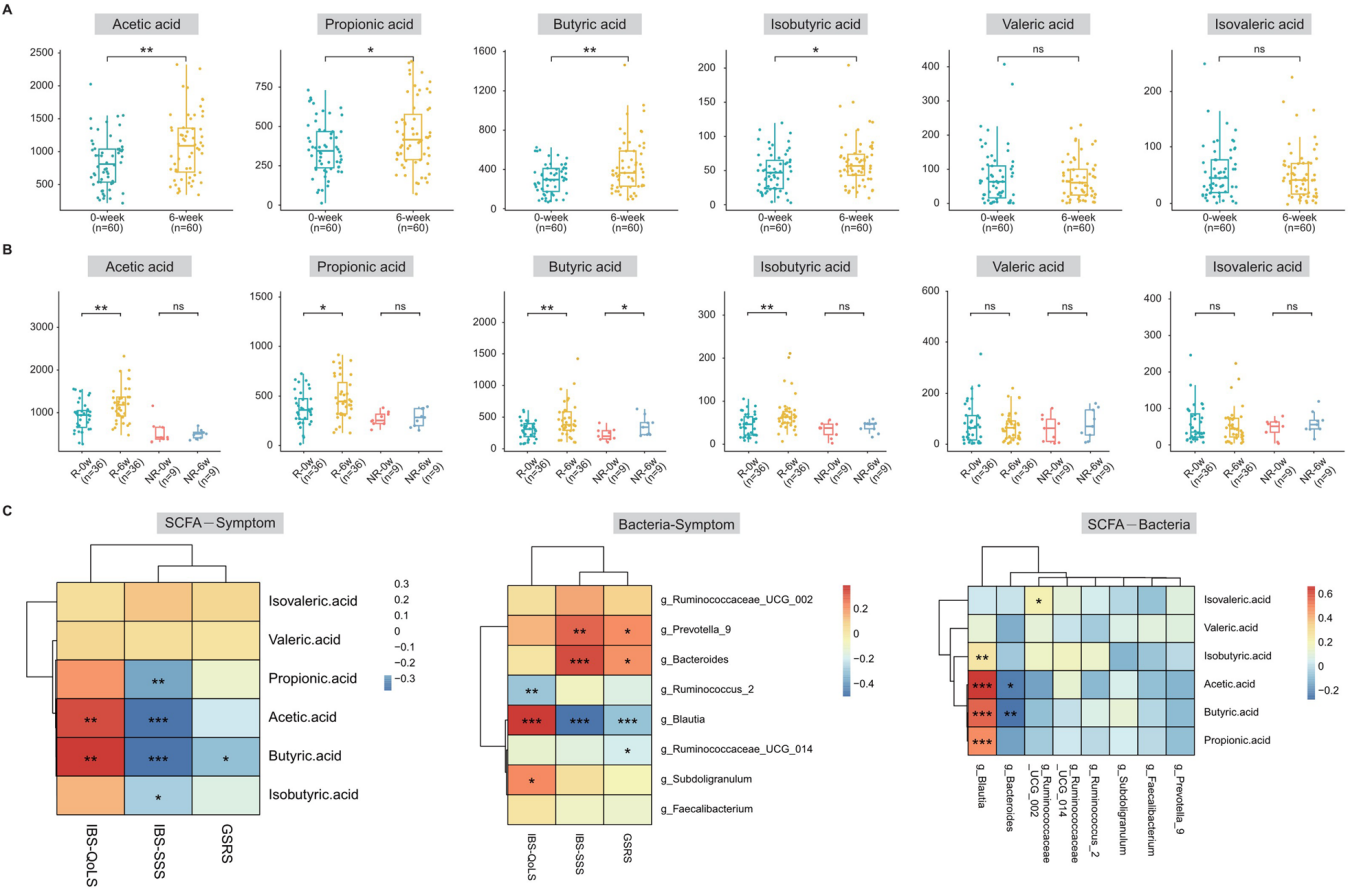
The levels of bacteria and their metabolites were positively correlated with clinical symptoms. **A** Concentrations of acetic acid, propionic acid, butyric acid, isobutyric acid, isovalerate acid, and valerate acid in the feces of IBS-C patients before and after linaclotide treatment. **B** Concentrations of acetic acid, propionic acid, butyric acid, isobutyric acid, isovalerate acid, and valerate acid in the feces of relief and no-relief IBS-C patients after treatment. **C** Correlations among the *Blautia* abundance, differential short-chain fatty acid (SCFA) content and clinical symptoms

According to relevant studies, *Blautia* species are SCFA-producing bacteria. A metabolomic analysis of fecal samples revealed that the concentrations of acetic acid (p < 0.01), propionic acid (p < 0.05), butyric acid (p < 0.01) and isobutyric acid (p < 0.05) were significantly increased in the feces of IBS-C patients treated with linaclotide for 6 weeks (Fig. 5A). A comparison between the relief and no relief groups demonstrated significantly higher levels of acetic acid (p < 0.0001), propionic acid (p < 0.0001), and isobutyric acid (p < 0.001) in the relief group after the treatment (Fig. 5B). We then performed a correlation analysis to investigate the relationships among the abundance of *Blautia*, the content of SCFAs, and clinical symptoms. Interestingly, we found a positive relationship between the abundance of *Blautia* and the contents of acetic acid, propionic acid, and butyric acid. Increased IBS-QoLS and decreased IBS-SSS and GSRS scores were positively correlated with the *Blautia* abundance. Additionally, increased IBS-QoLS and decreased IBS-SSS scores were positively correlated with the contents of acetic acid and butyric acid (Fig. 5C). These findings suggest that *Blautia* may play a crucial role in the efficacy of linaclotide treatment.

## Discussion

In this study, we demonstrated that linaclotide can improve defecation, relieve abdominal symptoms, and modify bowel evacuation habits in most IBS-C patients, and diarrhea is a common side effect. These results align with previous phase III clinical findings in China [16]. However, the results revealed individual differences in treatment effectiveness, which can potentially be attributed to the multifactorial nature of the pathophysiology. Dysbiosis of the gut microbiota has been identified as an important pathogenic factor in IBS-C patients [28]. Moreover, accumulating evidence has indicated that patients with IBS-C have intestinal microecological imbalances [29]. For example, IBS-C patients exhibit a higher gut microbial diversity and have a higher relative abundance of stool methanogens, predominantly *Methanobrevibacter* [17]. Although the mechanism through which intestinal microecological imbalance leads to IBS-C has not been fully elucidated, this imbalance possibly involves an excessively long retention time of feces in the intestinal tract, which changes the quantity and balance of intestinal microbes and the metabolites of the flora (SCFAs), the cellular components of the bacteria (lipopolysaccharides), or the interactions between the bacteria and the host immune system, all of which affect a variety of intestinal functions [30, 31].

Hence, we performed 16S sequencing on the stool of IBS-C patients collected before and after treatment for 6 weeks. The results showed that linaclotide could affect the intestinal flora, and our study also showed an increased abundance of *Blautia* and *Fusicatenibacter* in IBS-C patients after linaclotide treatment, even after adjusting for age, diet, and other factors. Recently, Tomita et al. reported “a significant reduction in the abundance of *Fusicatenibacter* in CD patients with IBS-D-like symptoms [32].” However, because the abundance of *Fusicatenibacter* was low and no significant enrichment of bacteria after linaclotide treatment was observed via LEfSe analysis, we mainly analyzed *Blautia*. A phase II clinical study recently found that MRx1234, which contains a strain of *Blautia hydrogenotrophica*, could improve the abdominal pain score and significantly improve the bowel habit rate of IBS-C patients [33]. In particular, we discovered that IBS-C patients exhibited a lower abundance of *Blautia* than healthy volunteers did, which illustrated that *Blautia* has the potential to become a novel, safe treatment option. As a member of the *Firmicutes* phylum, *Blautia* has shown promise in alleviating inflammatory and metabolic diseases because it has antibacterial activity against specific microorganisms [34, 35]. The composition and abundance of *Blautia* in the intestine are influenced by various factors, including diet, age, health, disease state, genotype, geography, and physiological conditions [36–38]. These findings align with the experimental results obtained in this study.

Moreover, the influence of the intestinal flora on dozens of clinical drugs has been revealed. A previous study highlighted the complex bidirectional interactions between the gut microbiota and various clinical drugs. The gut microbiome can be influenced by drugs, and vice versa, the gut microbiome can influence the treatment efficacy of drugs by impacting the drug structure and altering its bioavailability, biological activity or toxicity (pharmaceutical microbiology) [39]. In our study, we also found a significant increase in the *Blautia* abundance in the relief group but not in the no-relief group, indicating that linaclotide may modulate the *Blautia* abundance as part of its mechanism of action.

Previous studies have elucidated the role of *Blautia* as a commensal anaerobic bacterium that helps maintain the intestinal environmental balance, prevents inflammation, increases intestinal regulatory T cells, and produces SCFAs [40–42]. The total SCFA concentration in IBS-C patients was lower than that in healthy individuals, mainly due to reduced acetic acid and propionic acid levels [43]. In addition, the level of SCFAs is related to the viscosity of the stool of IBS patients [44]. If the stool texture of IBS-C patients (whose main clinical manifestation is constipation) is dry, the corresponding SCFA content also decreases. In our study, positive correlations were found among symptom improvement, the *Blautia* abundance, and the SCFA concentration, indicating that the therapeutic effect of linaclotide may involve increasing the SCFA concentrations through modulation of the intestinal flora, particularly by increasing the *Blautia* abundance. These results suggest that the gut microbiota and its metabolites may contribute to linaclotide treatment and its therapeutic effects (Additional file 6: Figure S6).

This study has several limitations. First, this study was not a randomized, controlled, double-blind clinical study, and the sample size was not sufficiently large. Second, gender based differences on IBS were clearly reported, and most IBS-C patients were female [45, 46]. Although the gender distribution of our patients showed the phenomenon above and we conduct a self-controlled pre-post study to exclude gender interference factor, we could not get valid results on gender differences in linaclotide treatment. Third, the effect of linaclotide on the intestinal flora has been reported only in China and has not been investigated in other regions. However, additional studies are needed to confirm this phenomenon.

## Conclusions

In summary, our study demonstrated that the gut microbiota may be not only necessary but also sufficient to mediate the effect of linaclotide in clinical settings. The efficacy of linaclotide is associated with modulation of the *Blautia* abundance and SCFA concentration. The abundance of *Blautia* after treatment could be used to predict the efficacy of linaclotide. Treatment with linaclotide supplemented with *Blautia* may be a potential method for improving the overall efficacy of clinical treatment for IBS-C.

### Supplementary Information

**Additional file 1: Figure S1.** Stacked bar chart. The taxonomic compositions of the 0-week and 6-week groups were compared at the phylum (**A**), class (**B**), order (**C**), family (**D**) and genus (**E**) levels.

**Additional file 2: Figure S2.** Clade evolution map of the 0-week and 6-week groups.

**Additional file 3: Figure S3.** Abundance of *Blautia* at each time point.

**Additional file 4: Figure S4.** Stacked bar chart. The taxonomic compositions of the relief and no relief groups were compared at the phylum (**A**), class (**B**), order (**C**), family (**D**), and genus (**E**) levels.

**Additional file 5: Figure S5.** Changes in alpha diversity indices (**A**) and principal coordinate analysis (PCoA) of the gut microbiota (**B**) of patients in the relief and no relief groups.

**Additional file 6: Figure S6.** Schematic illustration of the study.

**Additional file 7: Table S1. **Source of the patients.

**Additional file 8: Table S2.** Demographics of the normal population and IBS-C patients.

**Additional file 9: Table S3.** Nutrient intake in patients with different prognoses.

**Additional file 10: Table S4.** Comparison of the α diversity between before and after treatment.

**Additional file 11: Table S5.** Comparison of gut microbes at the phylum level between before and after treatment.

**Additional file 12: Table S6. **Comparison of gut microbes at the genus level between before and after treatment.

**Additional file 13: Table S7. **Comparison of gut microbes at the class level between before and after treatment.

**Additional file 14: Table S8. **Comparison of gut microbes at the order level between before and after treatment.

**Additional file 15: Table S9.** Comparison of gut microbes at the family level between before and after treatment.

## Data Availability

All the data generated or analyzed in the current study are included in this published article (and Additional files). Prof. Shiming Yang and Dr. Jianyun Zhou had full access to all the data in the study and take responsibility for the integrity of the data and the accuracy of the data analysis.

## Abbreviations

ANOSIM: Analysis of similarities
AUC: Area under the curve
BMI: Body mass index
BSFS: Bristol Stool Function Scale
CIC: Chronic idiopathic constipation
FDA: Food and drug administration
FFQ: Food frequency questionnaire
GSRS: Gastrointestinal symptom rating scale
IBS: Irritable bowel syndrome
IBS-C: Irritable bowel syndrome with constipation
IBS-D: Irritable bowel syndrome with diarrhea
IBS-QoLS: Quality of life scale of irritable bowel syndrome
IBS-SSS: Symptom severity score of irritable bowel syndrome
LDA: Linear discriminant analysis
LEfSe: LDA of effect size
NRS: Numeric Rating Scales
OTU: Operational taxonomic unit
PCoA: Principal coordinate analysis
SBMs: Spontaneous bowel movements
SCFA: Short-chain fatty acid

## References

[1] Lacy BE, Chey WD, Lembo AJ (2015). New and emerging treatment options for irritable bowel syndrome. Gastroenterol Hepatol (NY).

[2] Endo Y, Shoji T, Fukudo S (2015). Epidemiology of irritable bowel syndrome. Ann Gastroenterol.

[3] Ford AC, Moayyedi P, Lacy BE (2014). American college of gastroenterology monograph on the management of irritable bowel syndrome and chronic idiopathic constipation. Am J Gastroenterol.

[4] Liu JJ, Brenner DM (2021). Review article: current and future treatment approaches for IBS with constipation. Aliment Pharmacol Ther.

[5] Camilleri M (2015). American college of gastroenterology monograph on the management of irritable bowel syndrome. Expert Opin Pharmacother.

[6] Fukudo S, Kaneko H, Akiho H (2015). Evidence-based clinical practice guidelines for irritable bowel syndrome. J Gastroenterol.

[7] Ford AC, Moayyedi P, Chey WD (2018). American college of gastroenterology monograph on management of irritable bowel syndrome. Am J Gastroenterol.

[8] Busby RW, Bryant AP, Bartolini WP (2010). Linaclotide, through activation of guanylate cyclase C, acts locally in the gastrointestinal tract to elicit enhanced intestinal secretion and transit. Eur J Pharmacol.

[9] Bryant AP, Busby RW, Bartolini WP (2010). Linaclotide is a potent and selective guanylate cyclase C agonist that elicits pharmacological effects locally in the gastrointestinal tract. Life Sci.

[10] Farmer AD, Ruffle JK, Hobson AR (2018). Linaclotide increases cecal pH, accelerates colonic transit, and increases colonic motility in irritable bowel syndrome with constipation. Neurogastroent Motil.

[11] Rao SSC, Xiang X, Yan Y (2020). Randomised clinical trial: linaclotide vs placebo-a study of bi-directional gut and brain axis. Aliment Pharmacol Ther.

[12] Rao SSC, Quigley EMM, Shiff SJ (2014). Effect of Linaclotide on severe abdominal symptoms in patients with irritable bowel syndrome with constipation. Clin Gastroenterol Hepatol.

[13] Chey WD, Sayuk GS, Bartolini W (2021). Randomized trial of 2 delayed-release formulations of linaclotide in patients with irritable bowel syndrome with constipation. Am J Gastroenterol.

[14] Sayuk GS (2012). Linaclotide: promising IBS-C efficacy in an era of provisional study endpoints. Am J Gastroenterol.

[15] Chang L, Lacy BE, Moshiree B (2021). Efficacy of linaclotide in reducing abdominal symptoms of bloating, discomfort, and pain: a phase 3B trial using a novel abdominal scoring system. Am J Gastroenterol.

[16] Yang Y, Fang J, Guo X (2018). Linaclotide in irritable bowel syndrome with constipation: a phase 3 randomized trial in China and other regions. J Gastroenterol Hepatol.

[17] Villanueva-Millan MJ, Leite G, Wang J (2022). Methanogens and hydrogen sulfide producing bacteria guide distinct gut microbe profiles and irritable bowel syndrome subtypes. Am J Gastroenterol.

[18] Yu T, Guo F, Yu Y (2017). Fusobacterium nucleatum promotes chemoresistance to colorectal cancer by modulating autophagy. Cell.

[19] Shin N-R, Lee J-C, Lee H-Y (2014). An increase in the Akkermansia spp. population induced by metformin treatment improves glucose homeostasis in diet-induced obese mice. Gut.

[20] Li Y, Hong G, Yang M (2020). Fecal bacteria can predict the efficacy of rifaximin in patients with diarrhea-predominant irritable bowel syndrome. Pharmacol Res.

[21] Nanto-Hara F, Kanemitsu Y, Fukuda S (2020). The guanylate cyclase C agonist Linaclotide ameliorates the gut–cardio–renal axis in an adenine-induced mouse model of chronic kidney disease. Nephrol Dial Transpl.

[22] Aziz I, Whitehead WE, Palsson OS (2020). An approach to the diagnosis and management of Rome IV functional disorders of chronic constipation. Expert Rev Gastroenterol Hepatol.

[23] McIntosh K, Reed DE, Schneider T (2017). FODMAPs alter symptoms and the metabolome of patients with IBS: a randomised controlled trial. Gut.

[24] Feng Q, Liang S, Jia H (2015). Gut microbiome development along the colorectal adenoma-carcinoma sequence. Nat Commun.

[25] Lacy BE, Lembo AJ, Macdougall JE (2014). Responders vs clinical response: a critical analysis of data from linaclotide phase 3 clinical trials in IBS-C. Neurogastroenterol Motil.

[26] Phillips CM, Shivappa N, Hébert JR (2018). Dietary inflammatory index and biomarkers of lipoprotein metabolism, inflammation and glucose homeostasis in adults. Nutrients.

[27] Herman J, Pokkunuri V, Braham L (2010). Gender distribution in irritable bowel syndrome is proportional to the severity of constipation relative to diarrhea. Gend Med.

[28] Pittayanon R, Lau JT, Yuan Y (2019). Gut microbiota in patients with irritable bowel syndrome-a systematic review. Gastroenterology.

[29] Brenner DM, Harris LA, Chang CH (2022). Real-world treatment strategies to improve outcomes in patients with chronic idiopathic constipation and irritable bowel syndrome with constipation. Am J Gastroenterol.

[30] Dimidi E, Christodoulides S (2017). Mechanisms of action of probiotics and the gastrointestinal microbiota on gut motility and constipation. Adv Nutr.

[31] Chey WD, Kurlander J, Eswaran S (2015). Irritable bowel syndrome: a clinical review. JAMA.

[32] Tomita T, Fukui H, Morishita D (2023). Diarrhea-predominant irritable bowel syndrome-like symptoms in patients with quiescent crohn's disease: comprehensive analysis of clinical features and intestinal environment including the gut microbiome, organic acids, and intestinal permeability. J Neurogastroenterol Motil.

[33] Quigley EMM, Markinson L, Stevenson A (2023). Randomised clinical trial: efficacy and safety of the live biotherapeutic product MRx1234 in patients with irritable bowel syndrome. Aliment Pharmacol Ther.

[34] Chakravarthy SK, Jayasudha R, Prashanthi GS (2018). Dysbiosis in the gut bacterial microbiome of patients with uveitis, an inflammatory disease of the eye. Indian J Microbiol.

[35] Khattab MSA, Abd El Tawab AM, Fouad MT (2017). Isolation and characterization of anaerobic bacteria from frozen rumen liquid and its potential characterizations. Int J Dairy Sci.

[36] Odamaki T, Kato K, Sugahara H (2016). Age-related changes in gut microbiota composition from newborn to centenarian: a cross-sectional study. BMC Microbiol.

[37] Nakayama J, Watanabe K, Jiang J (2015). Diversity in gut bacterial community of school-age children in Asia. Sci Rep-Uk.

[38] Mao B, Gu J, Li D (2018). Effects of different doses of fructooligosaccharides (fos) on the composition of mice fecal microbiota, especially the *Bifidobacterium* composition. Nutrients.

[39] Maier L, Typas A (2017). Systematically investigating the impact of medication on the gut microbiome. Curr Opin Microbiol.

[40] Kim CH, Park J, Kim M (2014). Gut Microbiota-derived short-chain fatty acids, t cells, and inflammation. Immune Netw.

[41] Zhang C, He X, Sheng Y (2020). Allicin-induced host-gut microbe interactions improve energy homeostasis. FASEB J.

[42] Liu X, Mao B, Gu J, Chen W (2021). *Blautia*-a new functional genus with potential probiotic properties?. Gut Microbes.

[43] Gargari G, Taverniti V, Gardana C (2018). Fecal clostridiales distribution and short-chain fatty acids reflect bowel habits in irritable bowel syndrome. Environ Microbiol.

[44] Ringel-Kulka T, Choi CH, Temas D (2015). Altered colonic bacterial fermentation as a potential pathophysiological factor in irritable bowel syndrome. Am J Gastroenterol.

[45] Palsson OS, Whitehead W, Törnblom H (2020). Prevalence of Rome IV functional bowel disorders among adults in the United States, Canada, and the United Kingdom. Gastroenterology.

[46] Pecyna P, Gabryel M, Mankowska-Wierzbicka D (2023). Gender influences gut microbiota among patients with irritable bowel syndrome. Int J Mol Sci.

