# Supplementary figures and images for: The gut microbiota participates in the effect of linaclotide in patients with irritable bowel syndrome with constipation (IBS-C): a multicenter, prospective, pre-post study
Source: J Transl Med. 2024 Jan 23;22:98. doi: 10.1186/s12967-024-04898-1 (PMC10807057; doi:10.1186/s12967-024-04898-1)

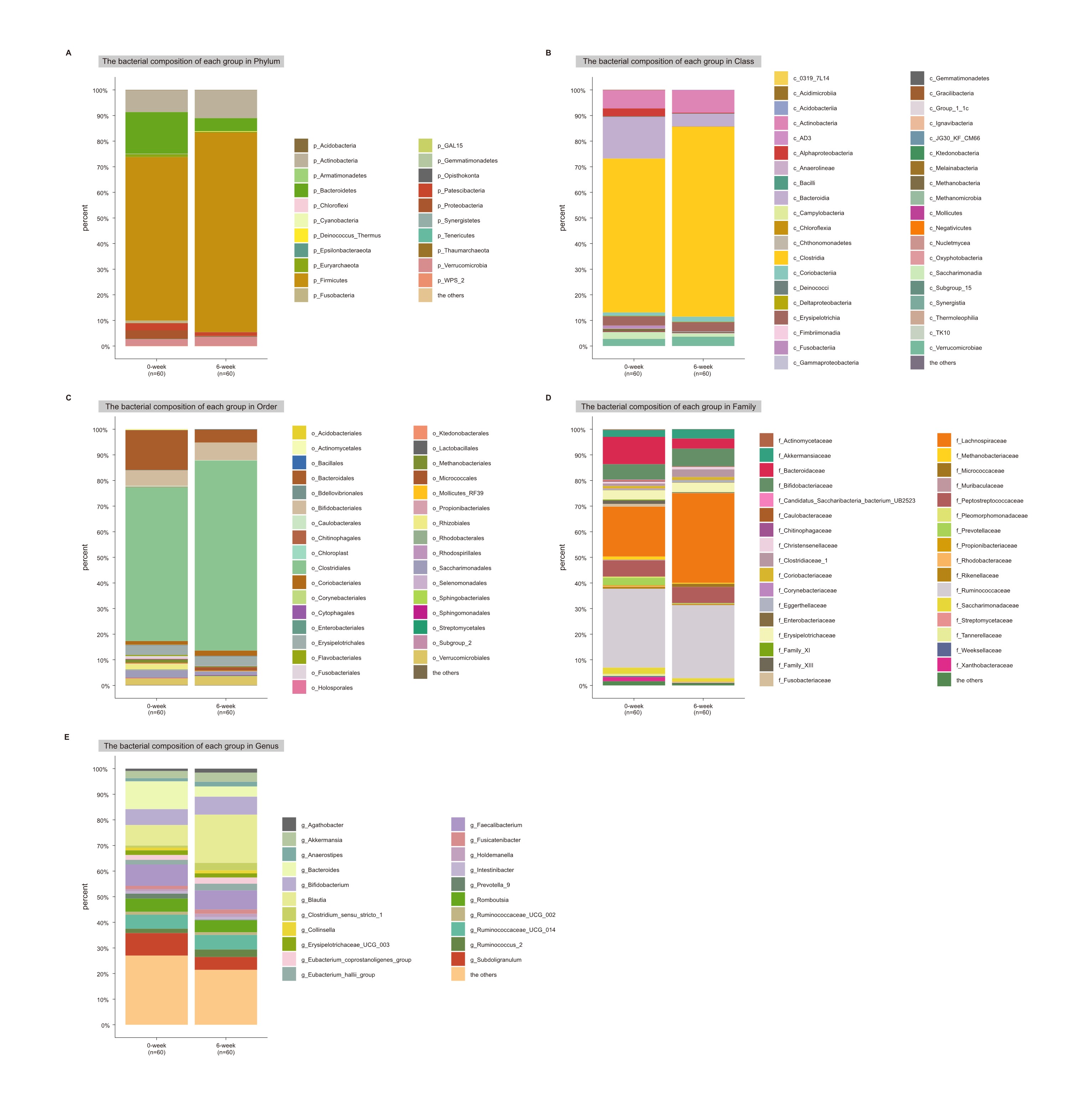

Supplement: Supplementary file 1 — Additional file 1: Figure S1. Stacked bar chart. The taxonomic compositions of the 0-week and 6-week groups were compared at the phylum (A), class (B), order (C), family (D) and genus (E) levels. [file 12967_2024_4898_MOESM1_ESM.jpg]

Figure S3

g\_Blautia

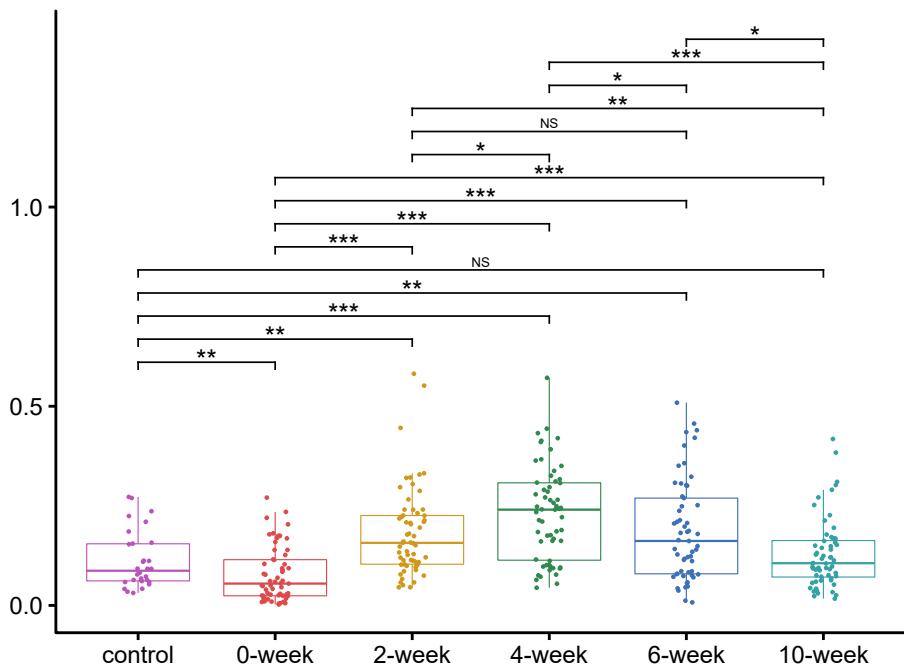

Supplement: Supplementary file 3 — Additional file 3: Figure S3. Abundance of Blautia at each time point. [file 12967_2024_4898_MOESM3_ESM.pdf]

A

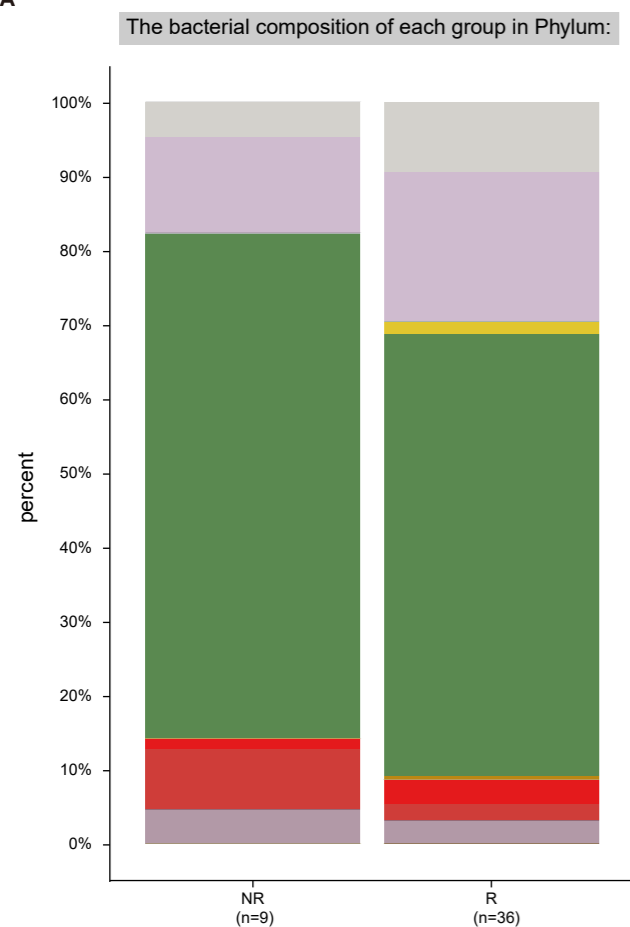

B

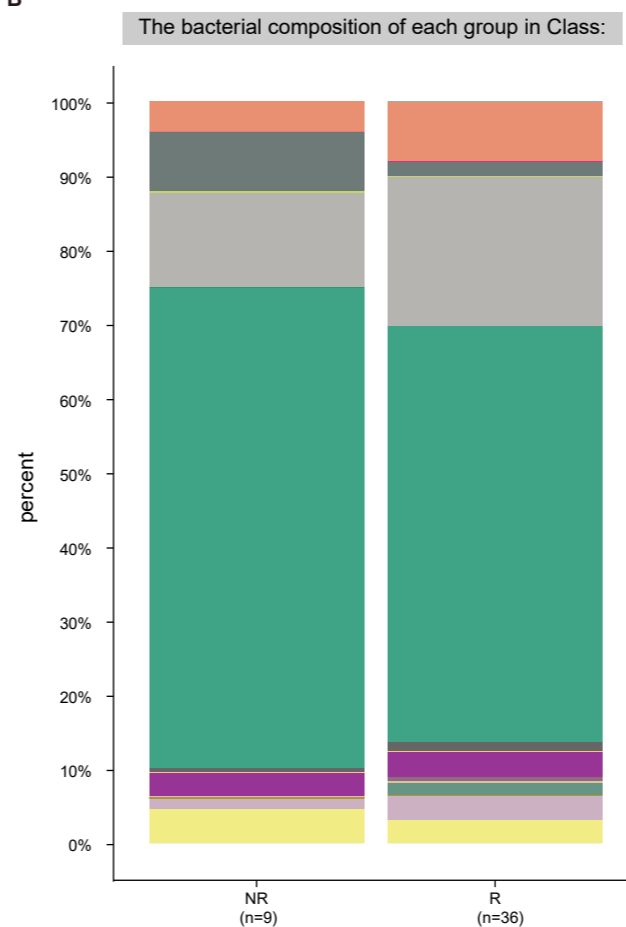

C

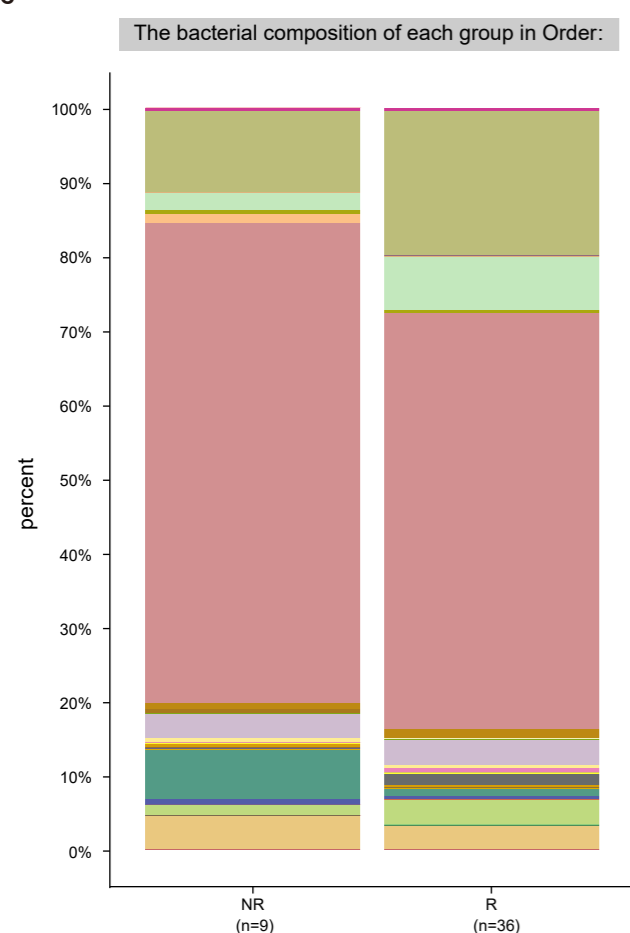

D

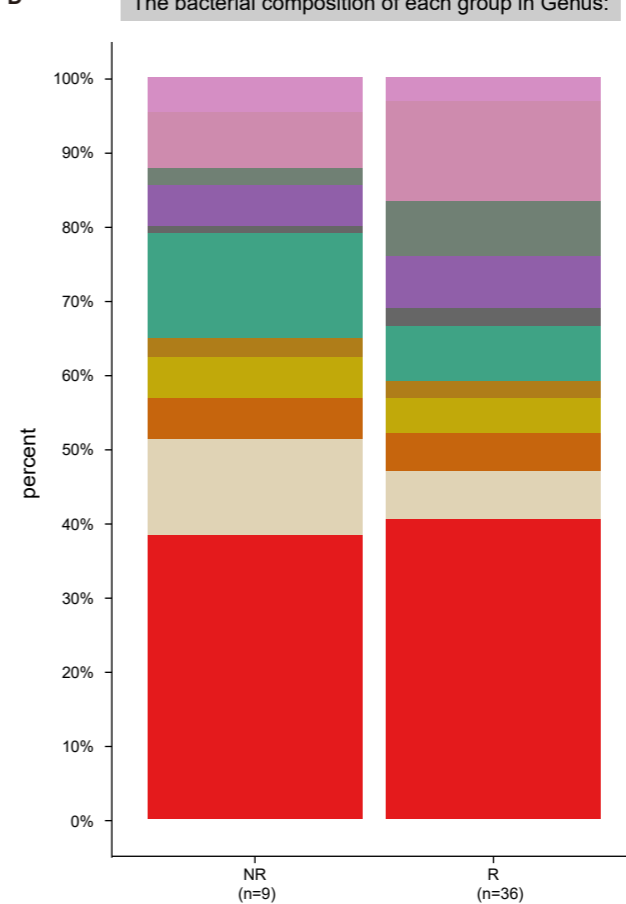

E

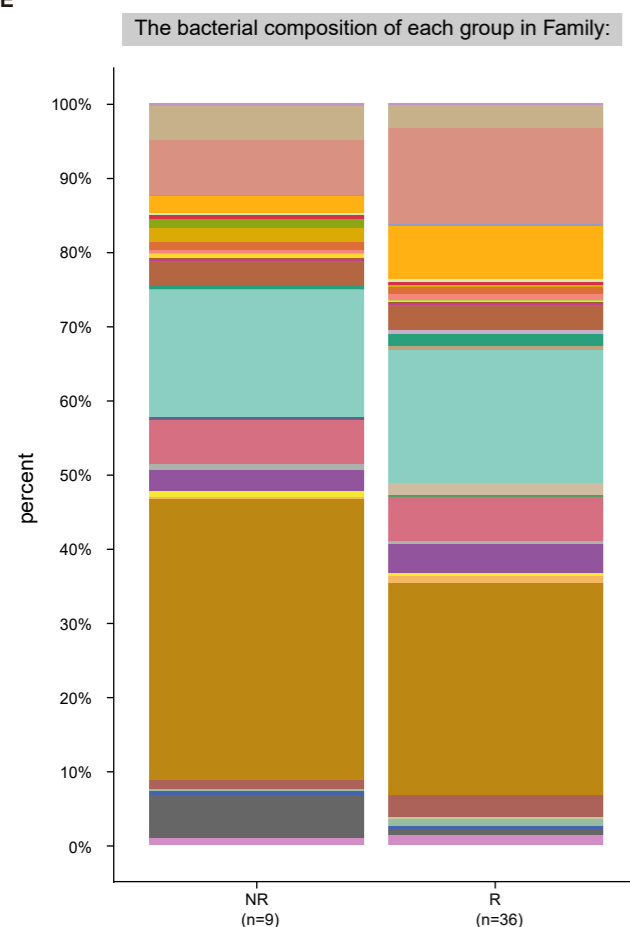

Supplement: Supplementary file 4 — Additional file 4: Figure S4. Stacked bar chart. The taxonomic compositions of the relief and no relief groups were compared at the phylum (A), class (B), order (C), family (D), and genus (E) levels. [file 12967_2024_4898_MOESM4_ESM.pdf]

**A**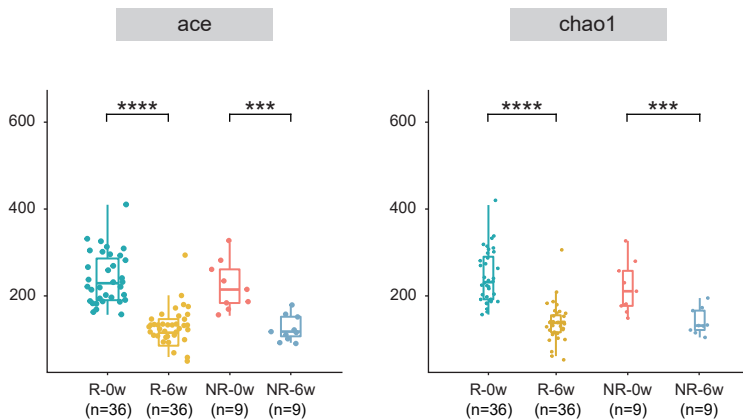**B**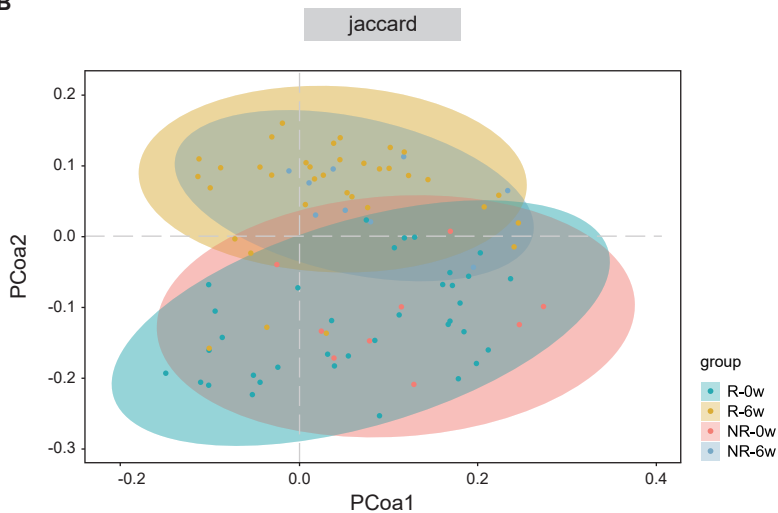

Supplement: Supplementary file 5 — Additional file 5: Figure S5. Changes in alpha diversity indices (A) and principal coordinate analysis (PCoA) of the gut microbiota (B) of patients in the relief and no relief groups. [file 12967_2024_4898_MOESM5_ESM.pdf]
