# Supplementary material for: The gut microbiota participates in the effect of linaclotide in patients with irritable bowel syndrome with constipation (IBS-C): a multicenter, prospective, pre-post study
Source: J Transl Med. 2024 Jan 23;22:98. doi: 10.1186/s12967-024-04898-1 (PMC10807057; doi:10.1186/s12967-024-04898-1)
Supplement: Supplementary file 2 — Additional file 2: Figure S2. Clade evolution map of the 0-week and 6-week groups. [file 12967_2024_4898_MOESM2_ESM.pdf]

Figure S2

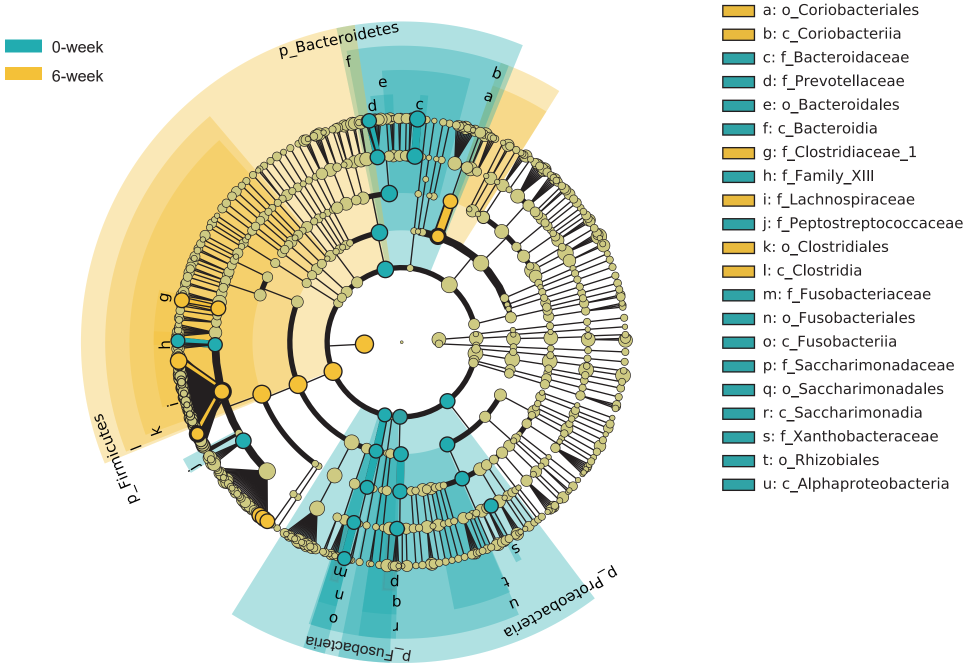
0-week
6-week
p_Bacteroidetes
p_Firmicutes
p_Fusobacteria
p_Proteobacteria
a: o_Coriobacteriales
b: c_Coriobacteriia
c: f_Bacteroidaceae
d: f_Prevotellaceae
e: o_Bacteroidales
f: c_Bacteroidia
g: f_Clostridiaceae_1
h: f_Family_XIII
i: f_Lachnospiraceae
j: f_Peptostreptococcaceae
k: o_Clostridiales
l: c_Clostridia
m: f_Fusobacteriaceae
n: o_Fusobacteriales
o: c_Fusobacteriia
p: f_Saccharimonadaceae
q: o_Saccharimonadales
r: c_Saccharimonadia
s: f_Xanthobacteraceae
t: o_Rhizobiales
u: c_Alphaproteobacteria
