# Supplementary material for: The gut microbiota participates in the effect of linaclotide in patients with irritable bowel syndrome with constipation (IBS-C): a multicenter, prospective, pre-post study
Source: J Transl Med. 2024 Jan 23;22:98. doi: 10.1186/s12967-024-04898-1 (PMC10807057; doi:10.1186/s12967-024-04898-1)
Supplement: Supplementary file 6 — Additional file 6: Figure S6. Schematic illustration of the study. [file 12967_2024_4898_MOESM6_ESM.pdf]

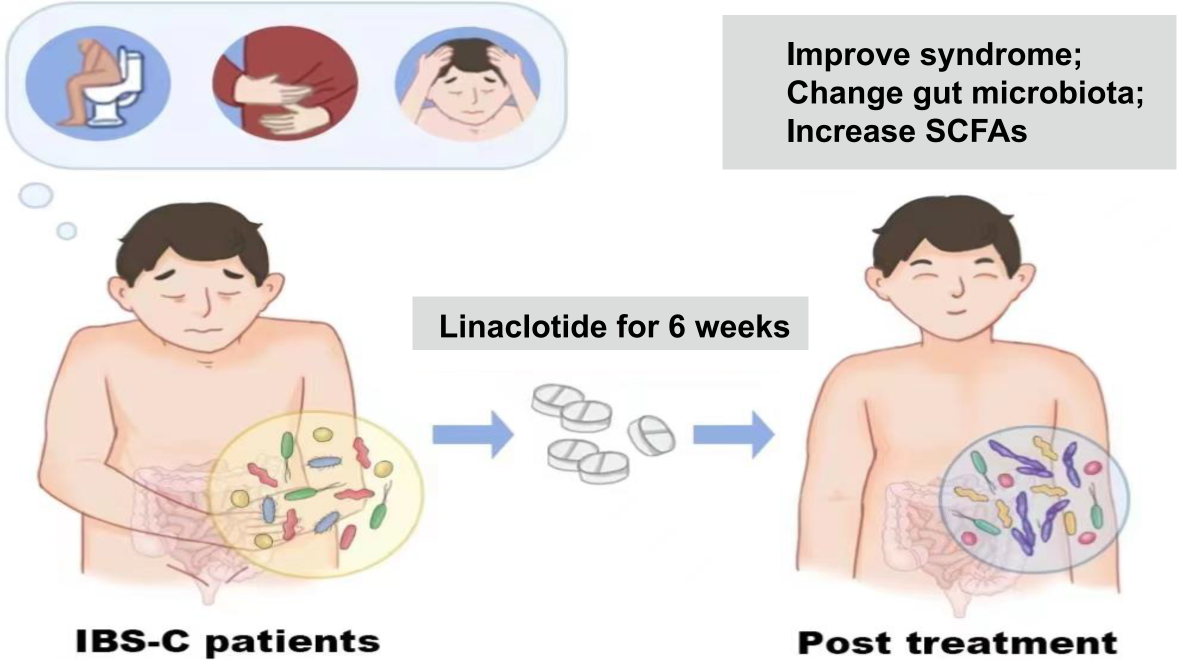
Improve syndrome;
Change gut microbiota;
Increase SCFAs
Linaclotide for 6 weeks
IBS-C patients
Post treatment
