# Supplementary material for: The gut microbiota participates in the effect of linaclotide in patients with irritable bowel syndrome with constipation (IBS-C): a multicenter, prospective, pre-post study
Source: J Transl Med. 2024 Jan 23;22:98. doi: 10.1186/s12967-024-04898-1 (PMC10807057; doi:10.1186/s12967-024-04898-1)
Supplement: Supplementary file 7 — Additional file 7: Table S1. Source of the patients. [file 12967_2024_4898_MOESM7_ESM.docx]

**Table S1:** The patient source

| Name of Hospital | Number of patients enrolled |
| --- | --- |
| The Second Affiliated Hospital of Army Medical University | 29 |
| Chonggang General Hospital | 1 |
| Suining Central Hospital | 8 |
| The First Affiliated Hospital of Chongqing Medical University Jinshan Hospital | 16 |
| Chongqing Ninth People's Hospital | 3 |
| Jiangjin District People's Hospital | 3 |
| Total | 60 |
