# Supplementary material for: The gut microbiota participates in the effect of linaclotide in patients with irritable bowel syndrome with constipation (IBS-C): a multicenter, prospective, pre-post study
Source: J Transl Med. 2024 Jan 23;22:98. doi: 10.1186/s12967-024-04898-1 (PMC10807057; doi:10.1186/s12967-024-04898-1)
Supplement: Supplementary file 8 — Additional file 8: Table S2. Demographics of the normal population and IBS-C patients. [file 12967_2024_4898_MOESM8_ESM.docx]

Table S2: The demographics between normal population and IBS-C patients

|  |  | Control (n=30) | IBS-C (n=60) | P value |
| --- | --- | --- | --- | --- |
| Gender | Female | 52(86.7) | 25(83.3) | 0.672 |
|  | Male | 8(13.3) | 5(16.7) |  |
| Age | Median (P25-P75) | 47(36.5~51.75) | 41.5(37.75~47. 25) | 0.284 |
|  | Mean±SD | 45.2±10.97 | 43.2±6.85 |  |
| BMI | Median (P25-P75) | 22.48(21.26~23.99) | 22.26(20.26~24.74) | 0.966 |
|  | Mean±SD | 22.62±2.76 | 22.74±3.19 |  |
| Education | Below college | 38(63.3) | 13(46.7) | 0.131 |
|  | College | 22(36.7) | 17(53.3) |  |
