# Supplementary material for: The gut microbiota participates in the effect of linaclotide in patients with irritable bowel syndrome with constipation (IBS-C): a multicenter, prospective, pre-post study
Source: J Transl Med. 2024 Jan 23;22:98. doi: 10.1186/s12967-024-04898-1 (PMC10807057; doi:10.1186/s12967-024-04898-1)
Supplement: Supplementary file 9 — Additional file 9: Table S3. Nutrient intake in patients with different prognoses. [file 12967_2024_4898_MOESM9_ESM.pdf]

Table S3: Nutrient intake in subjects with different prognoses

Time	Nutrients	Total(n=60)	Non-relief(n=9)	Relief(n=51)	P value
0-week	Energy/kJ	2804.72(1995.51~3458.21)	2597.16(1775.38~3009.11)	2844.95(2013.47~3684.64)	0.346
	Protein/g	122.44(74.21~164.21)	92.28(62.17~143.29)	130.92(74.9~177.45)	0.226
	Fat/g	118.84(76.89~159.54)	92.28(56.58~158.92)	122.79(81.68~165.83)	0.268
	Dietary fiber/g	14.16(8.89~22.38)	16.14(9.37~22.16)	13.77(8.44~22.46)	0.975
	Carbohydrate/g	254.46(180.72~363.37)	281.55(236.96~329.23)	243.48(157.43~406.15)	0.514
2-week	Energy/kJ	2722.08(2175.96~3310.79)	2560.6(2148.24~3688.23)	2723.42(2171.38~3112.62)	0.926
	Protein/g	124.16(82.39~159.01)	138.2(77.79~186.92)	123.39(82.11~144.17)	0.437
	Fat/g	123.43(79.84~143.18)	95.58(73.08~166.97)	123.47(81.5~140.2)	0.877
	Dietary fiber/g	14.67(9.1~19.17)	10.93(7.41~20.52)	14.72(10.54~19.23)	0.555
	Carbohydrate/g	281.1(207.77~375.86)	248.18(230.91~341.92)	293.74(198.47~381.93)	0.764
4-week	Energy/kJ	2990.01(2052.56~3753.94)	2611.59(1809.2~2990.01)	3281.52(2235.97~3789.78)	0.100
	Protein/g	128.39(80.63~182.56)	114.93(57.16~149.75)	130.56(85.56~185.98)	0.133
	Fat/g	125.51(83.28~179.75)	100.23(75.19~144.01)	130.56(84.85~183.97)	0.268
	Dietary fiber/g	15.95(9.94~24.29)	13.13(8.82~22.44)	17.86(10.86~27)	0.379
	Carbohydrate/g	343.77(205.64~467.34)	273.23(173.97~376.06)	365.8(199.43~509.59)	0.234
6-week	Energy/kJ	3108.22(2287.39~3944.82)	2250.68(1709.45~3096.49)	3300.5(2390.14~4284.37)	0.032
	Protein/g	127.66(83.71~159.09)	119.2(68.07~180.25)	128.01(87.04~159.43)	0.379
	Fat/g	121.22(84.1~159.56)	115.96(77.2~145.51)	128.01(85.83~163.61)	0.437
	Dietary fiber/g	15.41(10.25~24.02)	15.46(9.48~18.76)	15.35(10.41~28.97)	0.501
	Carbohydrate/g	334.81(218.33~507.25)	248.18(185.93~407.53)	371.95(218.25~535)	0.242
8-week	Energy/kJ	3008.63(2137.38~4131.96)	2465.68(1584.6~4151.71)	3243.91(2145.87~4144.42)	0.226
	Protein/g	125.78(91.56~165.65)	110.34(63.52~205.99)	129.43(91.67~164.81)	0.555
	Fat/g	140.02(91.86~196.09)	110.34(87.76~195.35)	142.15(91.56~199.26)	0.656
	Dietary fiber/g	15.38(11.08~22.7)	19.01(9.94~22.40)	15.31(11.34~23.01)	0.959
	Carbohydrate/g	292.18(209.51~393.43)	248.18(166.12~393.11)	293.71(208.72~394.05)	0.402
10-week	Energy/kJ	3341.85(2244.61~4017.73)	2649.78(1776.35~4063.81)	3507.5(2320.77~4021.04)	0.268
	Protein/g	151.99(102.66~191.59)	128.35(67.79~279.21)	158.57(106.57~192.04)	0.277
	Fat/g	142.32(105.49~177.16)	135.16(67.91~182.02)	155.74(106.84~178.47)	0.325
	Dietary fiber/g	16.36(9.76~29.03)	11.19(9.36~22.44)	17.43(9.76~29.36)	0.325
	Carbohydrate/g	338.46(225.82~455.52)	248.18(207.45~404.51)	368.54(230.22~486.02)	0.139
