# Supplementary material for: The gut microbiota participates in the effect of linaclotide in patients with irritable bowel syndrome with constipation (IBS-C): a multicenter, prospective, pre-post study
Source: J Transl Med. 2024 Jan 23;22:98. doi: 10.1186/s12967-024-04898-1 (PMC10807057; doi:10.1186/s12967-024-04898-1)
Supplement: Supplementary file 10 — Additional file 10: Table S4. Comparison of the α diversity between before and after treatment. [file 12967_2024_4898_MOESM10_ESM.docx]

**Table S4:** Comparison of α diversity before and after treatment

|  |  | 0 week | 6 weeks | P value | P (adjusted) |
| --- | --- | --- | --- | --- | --- |
| Ace | Median(P25-P75) | 237.77(192.99~268.39) | 130.44(110.1~144.88) | 0.000 | 0.013 |
|  | Mean±SD | 237.77±52.89 | 130.44±31.71 |  |  |
| Chao1 | Median(P25-P75) | 242.49(197~272.95) | 128.76(112.75~144.91) | 0.000 | 0.010 |
|  | Mean±SD | 242.49±55.09 | 128.76±33.47 |  |  |
| Faith_pd_ | Median(P25-P75) | 21.71(17.54~25.74) | 8.46(7.48~9.25) | 0.000 | 0.033 |
|  | Mean±SD | 22.54±6.37 | 8.61±2.03 |  |  |
| Observed_otus | Median(P25-P75) | 218.91(176.25~249.5) | 98.44(85.5~108.5) | 0.000 | P<0.001 |
|  | Mean±SD | 218.91±48.27 | 98.44±20.15 |  |  |
| Shannon | Median(P25-P75) | 5.06(4.61~5.54) | 5.01(4.74~5.41) | 0.000 | 0.459 |
|  | Mean±SD | 5.04±0.7 | 4.95±0.67 |  |  |
| Simpson | Median(P25-P75) | 0.92(0.88~0.95) | 0.93(0.9~0.96) | 0.045 | 0.601 |
|  | Mean±SD | 0.91±0.06 | 0.91±0.06 |  |  |
