# Supplementary material for: The gut microbiota participates in the effect of linaclotide in patients with irritable bowel syndrome with constipation (IBS-C): a multicenter, prospective, pre-post study
Source: J Transl Med. 2024 Jan 23;22:98. doi: 10.1186/s12967-024-04898-1 (PMC10807057; doi:10.1186/s12967-024-04898-1)
Supplement: Supplementary file 11 — Additional file 11: Table S5. Comparison of gut microbes at the phylum level between before and after treatment. [file 12967_2024_4898_MOESM11_ESM.pdf]

Table S5: Comparison of phylum level in gut microbiota before and after treatment

		0-week	6-week	P value	P (adjusted)
Euryarchaeota	Median(P25-P75)	0 (0~0)	0 (0~0)	0.206	0.356
	Mean±SD	0.01±0.05	0±0.01		
Acidobacteria	Median(P25-P75)	0 (0~0)	0 (0~0)	0.693	0.696
	Mean±SD	0±0	0±0		
Bacteroidetes	Median(P25-P75)	0.11(0.03~0.25)	0.02(0~0.05)	0.000	0.177
	Mean±SD	0.16±0.16	0.05±0.08		
Chloroflexi	Median(P25-P75)	0(0~0)	0(0~0)	0.000	0.811
	Mean±SD	0±0	0±0		
Firmicutes	Median(P25-P75)	0.66(0.52~0.8)	0.81(0.74~0.89)	0.000	P<0.001
	Mean±SD	0.63±0.2	0.78±0.16		
Patescibacteria	Median(P25-P75)	0(0~0.03)	0(0~0.01)	0.002	0.314
	Mean±SD	0.03±0.09	0.02±0.05		
Proteobacteria	Median(P25-P75)	0.01(0~0.03)	0(0~0)	0.001	0.480
	Mean±SD	0.04±0.09	0±0.01		
