# Supplementary material for: The gut microbiota participates in the effect of linaclotide in patients with irritable bowel syndrome with constipation (IBS-C): a multicenter, prospective, pre-post study
Source: J Transl Med. 2024 Jan 23;22:98. doi: 10.1186/s12967-024-04898-1 (PMC10807057; doi:10.1186/s12967-024-04898-1)
Supplement: Supplementary file 12 — Additional file 12: Table S6. Comparison of gut microbes at the genus level between before and after treatment. [file 12967_2024_4898_MOESM12_ESM.pdf]

Table S6: Comparison of genus level in gut microbiota before and after treatment

		0-week	6-week	P value	P (adjusted)
Bifidobacterium	Median(P25-P75)	0.02(0.01~0.06)	0.03(0.01~0.07)	0.208	0.105
	Mean ±SD	0.06 ±0.1	0.07 ±0.09		
Bacteroides	Median(P25-P75)	0.06(0.02~0.11)	0.01(0~0.04)	0.000	0.313
	Mean ±SD	0.1 ±0.12	0.04 ±0.07		
Anaerostipes	Median(P25-P75)	0.01(0~0.01)	0.02(0.01~0.02)	0.025	0.129
	Mean ±SD	0.01 ±0.02	0.02 ±0.01		
Blautia	Median(P25-P75)	0.06(0.02~0.11)	0.18(0.08~0.25)	0.000	0.003
	Mean ±SD	0.07 ±0.06	0.18 ±0.12		
Fusicatenibacter	Median(P25-P75)	0.01(0~0.01)	0.01(0.01~0.02)	0.035	P<0.001
	Mean ±SD	0.01 ±0.01	0.02 ±0.02		
Faecalibacterium	Median(P25-P75)	0.02(0.04~0.09)	0.02(0.05~0.09)	0.035	0.737
	Mean ±SD	0.08 ±0.11	0.07 ±0.07		
Subdoligranulum	Median(P25-P75)	0.06(0.02~0.1)	0.04(0.02~0.06)	0.047	0.240
	Mean ±SD	0.09 ±0.11	0.05 ±0.04		
Akkermansia	Median(P25-P75)	0(0~0.02)	0.01(0~0.04)	0.101	0.188
	Mean ±SD	0.03 ±0.07	0.04 ±0.09		
