# Supplementary material for: The gut microbiota participates in the effect of linaclotide in patients with irritable bowel syndrome with constipation (IBS-C): a multicenter, prospective, pre-post study
Source: J Transl Med. 2024 Jan 23;22:98. doi: 10.1186/s12967-024-04898-1 (PMC10807057; doi:10.1186/s12967-024-04898-1)
Supplement: Supplementary file 13 — Additional file 13: Table S7. Comparison of gut microbes at the class level between before and after treatment. [file 12967_2024_4898_MOESM13_ESM.pdf]

Table S7: Comparison of class level in gut microbiota before and after treatment

		0-week	6-week	P value	P (adjusted)
Actinobacteria	Mean ±SD	0.05 ±0.07	0.07 ±0.08	0.275	0.081
	Median(P25-P75)	0.03(0.01-0.05)	0.03(0.01-0.09)		
Coriobacteriia	Mean ±SD	0.01 ±0.02	0.02 ±0.03	0.007	0.669
	Median(P25-P75)	0.01(0-0.01)	0.01(0.01-0.02)		
Bacteroidia	Mean ±SD	0.13 ±0.14	0.05 ±0.08	<0.001	0.250
	Median(P25-P75)	0.06(0.02-0.22)	0.01(0-0.06)		
Bacilli	Mean ±SD	0.06 ±0.12	0.07 ±0.14	0.724	0.041
	Median(P25-P75)	0.02(0.01-0.05)	0.02(0.01-0.03)		
Clostridia	Mean ±SD	0.45 ±0.19	0.63 ±0.19	<0.001	0.022
	Median(P25-P75)	0.46(0.34-0.6)	0.67(0.49-0.78)		
Erysipelotrichia	Mean ±SD	0.03 ±0.05	0.03 ±0.05	0.080	0.452
	Median(P25-P75)	0.01(0.01-0.04)	0.02(0.01-0.04)		
Negativicutes	Mean ±SD	0.02 ±0.03	0.01 ±0.02	0.001	0.256
	Median(P25-P75)	0.01(0-0.02)	0(0-0.01)		
Saccharimonadia	Mean ±SD	0.02 ±0.08	0.01 ±0.04	0.004	0.611
	Median(P25-P75)	0(0-0.01)	0(0-0)		
Alphaproteobacteria	Mean ±SD	0.02 ±0.07	0 ±0.01	<0.001	0.284
	Median(P25-P75)	0(0-0.01)	0(0-0)		
Gammaproteobacteria	Mean ±SD	0.13 ±0.16	0.07 ±0.11	0.006	0.388
	Median(P25-P75)	0.06(0.03-0.17)	0.02(0.01-0.08)		
Mollicutes	Mean ±SD	0.03 ±0.12	0.01 ±0.04	0.966	0.868
	Median(P25-P75)	0(0-0.01)	0(0-0.01)		
Verrucomicrobiae	Mean ±SD	0.02 ±0.06	0.03 ±0.08	0.691	0.802
	Median(P25-P75)	0(0-0.01)	0(0-0.02)		
