# Supplementary material for: The gut microbiota participates in the effect of linaclotide in patients with irritable bowel syndrome with constipation (IBS-C): a multicenter, prospective, pre-post study
Source: J Transl Med. 2024 Jan 23;22:98. doi: 10.1186/s12967-024-04898-1 (PMC10807057; doi:10.1186/s12967-024-04898-1)
Supplement: Supplementary file 14 — Additional file 14: Table S8. Comparison of gut microbes at the order level between before and after treatment. [file 12967_2024_4898_MOESM14_ESM.pdf]

Table S8: Comparison of order level in gut microbiota before and after treatment

		0-week	6-week	P value	P (adjusted)
Bifidobacteriales	Mean ±SD	0.04 ±0.07	0.06 ±0.08	0.177	0.041
	Median(P25-P75)	0.01(0.01-0.04)	0.02(0.01-0.08)		
Coriobacteriales	Mean ±SD	0.01 ±0.02	0.02 ±0.03	0.007	0.669
	Median(P25-P75)	0.01(0-0.01)	0.01(0.01-0.02)		
Bacteroidales	Mean ±SD	0.12 ±0.15	0.05 ±0.08	<0.001	0.314
	Median(P25-P75)	0.06(0.01-0.21)	0.01(0-0.06)		
Lactobacillales	Mean ±SD	0.06 ±0.12	0.06 ±0.12	0.861	0.029
	Median(P25-P75)	0.02(0-0.05)	0.01(0.01-0.03)		
Clostridiales	Mean ±SD	0.45 ±0.19	0.63 ±0.19	<0.001	0.022
	Median(P25-P75)	0.46(0.34-0.6)	0.67(0.49-0.78)		
Erysipelotrichales	Mean ±SD	0.03 ±0.05	0.03 ±0.05	0.080	0.452
	Median(P25-P75)	0.01(0.01-0.04)	0.02(0.01-0.04)		
Selenomonadales	Mean ±SD	0.02 ±0.03	0.01 ±0.02	0.001	0.256
	Median(P25-P75)	0.01(0-0.02)	0(0-0.01)		
Saccharimonadales	Mean ±SD	0.02 ±0.08	0.01 ±0.04	0.004	0.611
	Median(P25-P75)	0(0-0.01)	0(0-0)		
Betaproteobacteriales	Mean ±SD	0.03 ±0.05	0 ±0	<0.001	0.336
	Median(P25-P75)	0.01(0-0.02)	0(0-0)		
Enterobacteriales	Mean ±SD	0.08 ±0.14	0.06 ±0.11	0.494	0.097
	Median(P25-P75)	0.02(0.01-0.09)	0.02(0.01-0.07)		
Mollicutes RF39	Mean ±SD	0.03 ±0.12	0.01 ±0.04	0.966	0.868
	Median(P25-P75)	0(0-0.01)	0(0-0.01)		
Verrucomicrobiales	Mean ±SD	0.02 ±0.06	0.03 ±0.08	0.685	0.802
	Median(P25-P75)	0(0-0.01)	0(0-0.02)		
