# Supplementary material for: The gut microbiota participates in the effect of linaclotide in patients with irritable bowel syndrome with constipation (IBS-C): a multicenter, prospective, pre-post study
Source: J Transl Med. 2024 Jan 23;22:98. doi: 10.1186/s12967-024-04898-1 (PMC10807057; doi:10.1186/s12967-024-04898-1)
Supplement: Supplementary file 15 — Additional file 15: Table S9. Comparison of gut microbes at the family level between before and after treatment. [file 12967_2024_4898_MOESM15_ESM.pdf]

Table S9: Comparison of family level in gut microbiota before and after treatment

		0-week	6-week	P value	P (adjusted)
Bifidobacteriaceae	Mean ±SD	0.04 ±0.07	0.06 ±0.08	0.177	0.041
	Median(P25-P75)	0.01(0.01-0.04)	0.02(0.01-0.08)		
Bacteroidaceae	Mean ±SD	0.08 ±0.11	0.04 ±0.07	0.001	0.895
	Median(P25-P75)	0.04(0.01-0.1)	0.01(0-0.04)		
Prevotellaceae	Mean ±SD	0.02 ±0.07	0 ±0.01	0.001	0.084
	Median(P25-P75)	0(0-0.01)	0(0-0)		
Enterococcaceae	Mean ±SD	0.01 ±0.03	0.02 ±0.06	0.340	0.761
	Median(P25-P75)	0(0-0)	0(0-0)		
Leuconostocaceae	Mean ±SD	0.01 ±0.07	0.01 ±0.04	0.291	0.109
	Median(P25-P75)	0(0-0)	0(0-0)		
Streptococcaceae	Mean ±SD	0.02 ±0.05	0.02 ±0.06	0.796	0.153
	Median(P25-P75)	0.01(0-0.02)	0.01(0-0.01)		
Clostridiaceae 1	Mean ±SD	0.01 ±0.01	0.03 ±0.07	<0.001	0.439
	Median(P25-P75)	0(0-0.01)	0.01(0-0.02)		
Lachnospiraceae	Mean ±SD	0.15 ±0.11	0.3 ±0.15	<0.001	0.036
	Median(P25-P75)	0.12(0.06-0.19)	0.29(0.19-0.38)		
Peptostreptococcaceae	Mean ±SD	0.05 ±0.06	0.05 ±0.04	0.054	0.932
	Median(P25-P75)	0.02(0.01-0.05)	0.03(0.02-0.07)		
Ruminococcaceae	Mean ±SD	0.24 ±0.18	0.24 ±0.16	0.742	0.197
	Median(P25-P75)	0.2(0.09-0.33)	0.2(0.12-0.36)		
Erysipelotrichaceae	Mean ±SD	0.03 ±0.05	0.03 ±0.05	0.080	0.452
	Median(P25-P75)	0.01(0.01-0.04)	0.02(0.01-0.04)		
Veillonellaceae	Mean ±SD	0.02 ±0.03	0.01 ±0.02	0.002	0.334
	Median(P25-P75)	0(0-0.01)	0(0-0)		
Saccharimonadaceae	Mean ±SD	0.02 ±0.07	0.01 ±0.04	0.010	0.611
	Median(P25-P75)	0(0-0.01)	0(0-0)		
Burkholderiaceae	Mean ±SD	0.02 ±0.05	0 ±0	<0.001	0.329
	Median(P25-P75)	0.01(0-0.02)	0(0-0)		
Enterobacteriaceae	Mean ±SD	0.08 ±0.14	0.06 ±0.11	0.494	0.097
	Median(P25-P75)	0.02(0.01-0.09)	0.02(0.01-0.07)		
Mollicutes RF39	Mean ±SD	0.02 ±0.11	0.01 ±0.02	0.883	0.800
	Median(P25-P75)	0(0-0)	0(0-0)		
Akkermansiaceae	Mean ±SD	0.02 ±0.06	0.03 ±0.08	0.697	0.804
	Median(P25-P75)	0(0-0.01)	0(0-0.02)		
